# Inflammation-mediated fibroblast activation and immune dysregulation in collagen VII-deficient skin

**DOI:** 10.3389/fimmu.2023.1211505

**Published:** 2023-09-20

**Authors:** Morgan Anderson-Crannage, Alex M. Ascensión, Olga Ibanez-Solé, Hongwen Zhu, Edo Schaefer, Darcy Ottomanelli, Bruno Hochberg, Jian Pan, Wen Luo, Meijuan Tian, Yaya Chu, Mitchell S. Cairo, Ander Izeta, Yanling Liao

**Affiliations:** ^1^Department of Pediatrics, New York Medical College, Valhalla, NY, United States; ^2^Department of Cell Biology and Anatomy, New York Medical College, Valhalla, NY, United States; ^3^Biodonostia Health Research Institute, Tissue Engineering Group, San Sebastian, Spain; ^4^Department of Research & Development, Guizhou Atlasus Technology Co., Ltd., Guiyang, China; ^5^Department of Medicine, New York Medical College, Valhalla, NY, United States; ^6^Department of Pathology, Microbiology and Immunology, New York Medical College, Valhalla, NY, United States; ^7^Department of Biomedical Engineering and Science, School of Engineering, Tecnun University of Navarra, San Sebastian, Spain

**Keywords:** epidermolysis bullosa, inflammation, immune suppression, dermal microenvironment, fibroblast activation, interleukin-1, single-cell RNA sequencing, squamous cell carcinoma

## Abstract

Inflammation is known to play a critical role in all stages of tumorigenesis; however, less is known about how it predisposes the tissue microenvironment preceding tumor formation. Recessive dystrophic epidermolysis bullosa (RDEB), a skin-blistering disease secondary to *COL7A1* mutations and associated with chronic wounding, inflammation, fibrosis, and cutaneous squamous cell carcinoma (cSCC), models this dynamic. Here, we used single-cell RNA sequencing (scRNAseq) to analyze gene expression patterns in skin cells from a mouse model of RDEB. We uncovered a complex landscape within the RDEB dermal microenvironment that exhibited altered metabolism, enhanced angiogenesis, hyperproliferative keratinocytes, infiltration and activation of immune cell populations, and inflammatory fibroblast priming. We demonstrated the presence of activated neutrophil and Langerhans cell subpopulations and elevated expression of PD-1 and PD-L1 in T cells and antigen-presenting cells, respectively. Unsupervised clustering within the fibroblast population further revealed two differentiation pathways in RDEB fibroblasts, one toward myofibroblasts and the other toward a phenotype that shares the characteristics of inflammatory fibroblast subsets in other inflammatory diseases as well as the IL-1-induced inflammatory cancer-associated fibroblasts (iCAFs) reported in various cancer types. Quantitation of inflammatory cytokines indicated dynamic waves of IL-1α, TGF-β1, TNF, IL-6, and IFN-γ concentrations, along with dermal NF-κB activation preceding JAK/STAT signaling. We further demonstrated the divergent and overlapping roles of these cytokines in inducing inflammatory phenotypes in RDEB patients as well as RDEB mouse-derived fibroblasts together with their healthy controls. In summary, our data have suggested a potential role of inflammation, driven by the chronic release of inflammatory cytokines such as IL-1, in creating an immune-suppressed dermal microenvironment that underlies RDEB disease progression.

## Introduction

Recessive dystrophic epidermolysis bullosa (RDEB) is a devastating disease secondary to mutations in the *COL7A1* gene that encodes type VII collagen (C7) (1). Patients with RDEB suffer from recurrent blistering, chronic inflammation, fibrosis, and ulceration in the skin, gastrointestinal tract, and genitourinary tracts, resulting in progressive multisite debilitating deformities such as pseudosyndactyly. Cutaneous squamous cell carcinoma (cSCC) is the major lethal complication affecting patients with RDEB, most frequently arising within the areas of chronic nonhealing wounds in extremities (2, 3). The overall driver mutations in RDEB-associated cSCC are the same as those in spontaneous ultraviolet light- induced cSCC and head and neck SCC and include genes such as HRAS, NOTCH1, TP53, and CDKN2A; however, mutations in these genes occur much earlier in life in patients with EB (4–6).

Multiple lines of evidence demonstrate that the dermal microenvironment plays an important role in RDEB-cSCC development. Carcinogen-treated C7 hypomorphic (C7^hypo^) mice (an animal model of RDEB that expresses about 10% of normal C7 level (7)) developed invasive tumors reminiscent of RDEB-cSCC, whereas wild-type (WT) mice formed benign papillomas (8). Excessive TGF-β signaling, tissue stiffening, and dysregulated mechanosensing in part contribute to the aggressiveness of RDEB-cSCC. Furthermore, previous studies demonstrated a resemblance between the fibroblasts derived from RDEB patients without a clinical diagnosis of cSCC and the spontaneous cancer-associated fibroblasts (CAFs) (9).

Single-cell RNA sequencing (scRNAseq) analysis across multiple cancer types and species revealed that CAFs are composed of spatially distinct subpopulations with a postulated “interconvertible” or “push-pull” dynamic over the course of tumor development (10–15). Although the phenotypes and nomenclature for the CAF subpopulations varied in the literature, “inflammatory” or “immunomodulatory” CAFs (iCAF) with increased expression of inflammatory cytokines and “mechanoresponsive” or “myofibroblastic” CAFs (myCAFs) with high levels of α-smooth muscle actin (αSMA) and other contractile proteins have consistently been reported. Tumor-secreted TGF-β1 and IL-1 are the key cytokines responsible for the induction of myCAF and iCAF phenotypes, respectively, in pancreatic ductal adenocarcinoma (15). Specifically, IL-1α induces the iCAF phenotype by activating nuclear factor kappa-light-chain-enhancer of activated B cell (NF-κB) signaling and leukemia inhibitory factor (LIF) expression, subsequently activating the JAK/STAT pathway. Accordingly, the IL-1 receptor (IL-1R1) was significantly upregulated in iCAFs compared with myCAFs. Meanwhile, TGF-β signaling antagonizes IL-1α-induced-iCAF by downregulating IL-1R1 and promoting myofibroblast differentiation (15).

scRNAseq has also revealed intra- and intertissue fibroblast heterogeneity under both normal and perturbed conditions (11, 16–18), which has suggested immune effector functions in innate host defense and inflammation (19).

Inflammation-induced fibrosis is the final pathological phase in chronic autoimmune diseases; end-state liver, kidney, and heart disease; and idiopathic pulmonary fibrosis (20). There is also clear evidence for the relevance of inflammation in the pathological progression and disease complications of RDEB. Several studies have reported aberrant immune cell profiles in skin wounds and an increased presence of proinflammatory cytokines and chemokines in the plasma and blister fluid of patients with RDEB (21–25). Fibroblasts derived from RDEB chronic wounds exhibited increased secretion of IL-6 and IL-1β compared with control fibroblasts and fibroblasts derived from nonlesioned RDEB skin (26). Moreover, the plasma IL-6/IL-10 ratio correlates with the severity of RDEB manifestation (25). Circulating IL-1β has also been found to be elevated in a patient with RDEB, and treatment with an anti-IL-1β monoclonal antibody significantly improved wound healing and reduced new blister formation; however, its efficacy waned over time (27). Inflammatory cytokines, including IL-6 were also found to be upregulated in C7^hypo^ and *Col7a1^−/−^* (knockout (KO)) mouse models of RDEB (28, 29). Importantly, recent studies demonstrated that the most significant molecular changes in C7^hypo^ mouse skin during progressive fibrotic development were related to inflammatory responses (30). Moreover, administration of an anti-inflammatory heptapeptide Ang-(1-7) in RDEB mice significantly attenuated the progression of fibrosis and improved their survival (30). Reciprocally, treatment of C7^hypo^ mice with losartan, an antifibrotic agent, resulted in a suppression of their inflammatory responses (28). These studies highlighted an intricate interplay between chronic inflammatory and fibrosis in RDEB.

In this study, we performed transcriptome analysis of single cells isolated from the KO mice at less than 2 weeks of age, when the TGF-β-induced fibrotic signaling has not been fully activated (29). We demonstrate that while the mice are in an early phase of disease progression, their skin exhibits features of protumor inflammation and inflammatory fibroblast activation. Through time-course profiling of cytokines using both KO and C7^hypo^ mouse models, we report multiple inflammatory cascades likely orchestrated by IL-1α. We further validate the effects of IL-1α and other cytokines on the inflammatory phenotypes of RDEB mouse and human RDEB patient-derived dermal fibroblasts.

## Materials and methods

### Mice

Two mouse models of RDEB were utilized in this study. C57BL6/J *Col7a1*^+/−^ mice, kindly provided by Jouni Uitto, MD, at Jefferson Medical College, were developed by targeted ablation of the *COL7A1* gene through out-of-frame deletion of exons 14–18 (31). Breeding of this mouse strain gives rise to *Col7a1*^−/−^ (KO) mice. KO mice have a median life span of 2 days without treatment and rarely survive past 3 weeks of age (32). *Col7a1^fNeo^* mice, kindly provided by Alexander Nyström at the University of Heidelberg, were developed on amixed C57BL/6 129sv background by replacing an 11-kb genomic fragment spanning exon 2 of *Col7a1* with a targeting construct containing a phosphoglycerate kinase promoter-driven neomycin phosphotransferase (PGK-Neo) expression cassette (7). Breeding of this mouse strain gives rise to C7^hypo^ mice. C7^hypo^ mice have an improved median life span (around 12 days) than the KO mice and develop mitten deformities in long-term survived mice. Both RDEB mouse models developed blisters at birth and were validated for their genotype by PCR. All animal studies were conducted using protocols approved by the New York Medical College Institutional Animal Care and Use Committee (IACUC).

### Cell lines and culture

To derive mouse fibroblasts, paw skin was dissected from WT and KO mice and digested in 1% dispase overnight at 4˚C. The dermis was then separated from the epidermis, cut into small pieces, and cultured in a fibroblast medium for the outgrowth of fibroblasts. Human RDEB-specific fibroblasts were derived from the nonlesional, noninflamed skin of six individuals with RDEB after informed, written consent. The demographics of RDEB patient donors are described in **Table 1** (29, 33, 34). The normal control fibroblasts included foreskin fibroblasts (NC1) (33) and two adult dermal fibroblast lines (NC2, cat # 106-05A, Sigma-Aldrich and NC3, cat # PCS-201-012, ATCC). Fibroblasts were maintained in low-glucose Dulbecco’s modified Eagle’s medium (DMEM) supplemented with 10% FBS. For cytokine stimulation, each cell line was seeded in duplicate wells of six- or 12-well culture plates (Corning, Lowell, MA, USA) and cultured for 24 h. The medium was then replaced with a low-glucose DMEM medium (basal medium) for 24 h. For the human fibroblasts, recombinant human IL-1α (Biolegend, San Diego, CA, USA), IL-6 (R&D, Minneapolis, MN, USA), TNF (Biolegend, San Diego, CA, USA), IFN-γ (Biolegend), or TGF-β1 (R&D, Minneapolis, MN, USA) was added to a final concentration of 4 ng/mL, 2 ng/mL, 100 ng/mL, 100 ng/mL, and 5 ng/mL to the basal coculture medium, respectively. For the mouse fibroblasts, recombinant mouse IL-1α (Biolegend), TNF Biolegend, San Diego, CA, USA), IFN-γ (Biolegend, San Diego, CA, USA), or TGF-β1 (Biolegend, San Diego, CA, USA) was added to a final concentration of 4 ng/mL, 100 ng/mL, 100 ng/mL, and 5 ng/mL to the basal coculture medium, respectively. Cytokine treatment concentrations were chosen either based on previous literature (29) or from the lowest concentration needed to elicit the same maximal response of PDPN expression among each fibroblast cell line independently. At the end of the 24-h coculture, the media was collected for ELISA quantification of sST2, and the cells were trypsinized with 200 µL of TrypLE and washed with Magnetic-activated cell sorting (MACS) rinsing buffer. Collected cells were immediately stained for flow cytometry analysis.

**Table 1:** Demographics of RDEB patient donors for fibroblast derivation.

	Age	Sex	Mutations
EB1	13 months	Male	Compound heterozygous premature stop codon mutations in exon 5 (c609delC) and exon 75 (c6269delC) (28)
EB2	1.5 years	Male	Homozygous mutation designated c2305-14del10ins2 interrupting the splice junction of exon 17 with the 5′ splicing site of intron 17 (28)
EB3	2.5 years	Male	Compound heterozygous mutations c.2005c->t (causing a premature stop codon in exon 15) and c.7318g->c (causing formation of an arginine codon from a glycine codon in exon 95) (29)
EB4	4.5 years	Male	Homozygous mutation (c.2035_14del10Ins2) encompassing the splice junction of exon 17 and intron 17, resulting in the deletion of 5 and insertion of 2 amino acids (29).
EB5	22 years	Male	Homozygous mutation c425G>A in exon 3 resulting in out-of-frame transcripts (23)
EB6	40 years	Male	Compound heterozygote for an acceptor splice site mutation, IVS30-1G>A, and a nonglycine missense substitution, c.6205C>T (exon 74; p.Arg2069Cys) (23)

### Flow cytometry analysis

Each fibroblast sample was blocked in MACS staining buffer and subsequently stained with PDPN (PE) and ST2 (647)-conjugated antibodies (Biolegend) for 30 min. Samples were washed and resuspended with 300 µL MACS rinsing buffer and immediately run on BD FACS Canto II Flow Cytometer. Using FCS Express 7 Flow, fibroblasts were gated from an FSC and SSC plot and measured for PE and 647 MFI to determine PDPN and membrane-bound ST2 expression, respectively.

### Cytokine quantitation

To quantitate the cytokines in the skin, paw skin lysate was prepared following lysis of stripped skin tissue in the presence of protease inhibitors (Cell Signaling Technology, Danvers, MA, USA) and homogenization in gentleMACS™ M tubes using the gentleMACS Dissociator (Miltenyi Biotec, Charlestown, MA, USA). Protein concentrations were determined using the Bio-Rad Protein Assay (Bio-Rad Laboratory, Hercules, CA, USA). Paw skin lysate samples were diluted in assay diluent from the appropriate assay kit to a consistent concentration across all samples, either to 10 µg or 100 µg per 50 µL or 100 µL, depending on cytokine abundance and assay type, using the total concentration measured by the Bio-Rad Protein Assay. Mouse IL-1α, IL-1β, and Th1/Th2/Th17 cytokines were quantitated using the CBA Flex set (BD Bioscience, Franklin Lakes, NJ, USA). Mouse IL-1ra and TGF-β1 were quantitated using an ELISA kit (R&D). Mouse IL-33, mouse ST2, and human ST2 were quantitated using an ELISA kit (Thermo Fisher Scientific, Wilmington, DE, USA). ELISA and CBA assays were performed according to the protocols provided by each manufacturer. Optical densities (OD) from ELISA assays were measured with the FilterMax F5 Multi-Mode Microplate Reader (Molecular Devices, San Jose, CA, USA). Cytokine concentration was determined by plotting the OD or median fluorescence intensity (MFI) of each sample on each cytokine concentration curve and multiplying the result by a dilution factor to give a sample concentration in picograms per milligram. Plasma was collected from blood in EDTA-containing tubes and diluted 1:2 in the assay diluent from the appropriate assay kit. Plasma cytokine concentration was measured the same as the lysate but expressed in picograms per milliliter of concentrations. Supernatant samples from cell culture were also diluted 1:2 in assay diluent and had cytokines quantitated by ELISA. Boxplots, dot plots, and trendlines were generated with the ggplot2 package in R.

### Immunohistochemical analysis

The collected tissues were embedded in Tissue-Tec OCT Compound and snap-frozen on dry ice. For each specimen, 6 µm serial sections were cut and stored at −80°C. Sections were fixed in freshly diluted 4% paraformaldehyde (Electron Microscopy Sciences, Hatfield, PA, USA) and blocked with M.O.M. blocking reagent (for antibodies raised in mice) or CAS-Block (for antibodies raised in other species of animals) with 0.1% Triton (Sigma, St. Louis, MO, USA). The slides were then incubated with primary and corresponding secondary antibodies and mounted in a Vectashield mounting medium containing DAPI (Vector Laboratories, Newark, CA, USA). Images were acquired using the EVOS M5000 imaging system (Thermo Fisher Scientific) or ZEISS LSM 980 plus confocal microscope using the same settings between the different groups in each set of experiments.

### Mouse paw skin single-cell isolation, library preparation, and scRNAseq

The skin was dissected from the paws of D11 WT and KO mice (*N* = 2), cut into small pieces, and dissociated into a single-cell suspension using a combination of enzymatic digestion and mechanical dissociation. Briefly, tissues were digested using the components in Multi Tissue Dissociation Kit 1 (Miltenyi Biotec), following the manufacturer’s recommendation for 5 h at 37°C. Samples were then transferred into gentleMACS™ C tubes and loaded onto the gentleMACS™ Octo Dissociator with Heaters (Miltenyi Biotec) with the program selected for skin dissociation. Cells were then passed through 70-μm and 40-μm filters (Falcon, Corning, NY, USA) sequentially. Dead cells were removed using Ficoll-Plaque PLUS (GE Healthcare, Chicago, IL, USA) separation. The viability of the cells was evaluated by Trypan blue staining. Library preparation and sequencing were done by Singulomics Corporation (https://singulomics.com/, Bronx, NY, USA). Viable cell suspensions were loaded into the Chromium Controller (10x Genomics, Pleasanton, CA, USA) to generate gel beads-in-emulsion (GEM) with each GEM containing a single cell as well as barcoded oligonucleotides. Next, the GEMs were placed in the SimpliAmp 96-well Thermal Cycler (Thermo Fisher Scientific, Wilmington, DE, USA), and reverse transcription was performed in each GEM (GEM-RT). After the reaction, the complementary cDNA was amplified and cleaned using Silane DynaBeads (10X Genomics, Pleasanton, CA, USA) and the SPRIselect Reagent kit (Beckman Coulter, Indianapolis, IN, USA). Amplified full-length cDNAs from poly-adenylated mRNA were then used to generate 5′ Gene Expression library (Chromium Next GEM Single Cell 5′ Reagent Kits v2 (Dual Index)), following the manufacturer’s instructions (10x Genomics, Pleasanton, CA, USA). Amplified cDNAs and the libraries were measured by the Qubit dsDNA HS assay (Thermo Fisher Scientific, Wilmington, DE, USA), and quality was assessed by the BioAnalyzer (Agilent Technologies, Santa Clara, CA, USA). Libraries were sequenced on a NovaSeq 6000 instrument (Illumina, San Diego, CA, USA), and reads were subsequently processed using the 10x Genomics Cell Ranger analytical pipeline (v6.0.1) and mouse reference genome mm10. Dataset aggregation was performed using the cellranger aggr function, normalizing for the total number of confidently mapped reads across libraries. A total of 14,856 cells were sequenced, with 24,774 postnormalization mean reads per cell. Methods for bioinformatic analysis, including basic processing, cell assignment algorithms, analysis of subtype cells, GO term scoring, and trajectory inferences are described in supplementary data.

### Ingenuity pathway analysis

Globally differentially expressed genes with a log2-fold change greater than 1.2 and an adjusted *p*-value of less than 0.05 were uploaded into Qiagen’s ingenuity pathway analysis (IPA) system for core analysis.

### Statistical analysis

The Chi-square test was used to determine the statistical significance of the quantities of each cell type between the WT and KO samples of the scRNAseq data. Statistical significance between groupings of WT vs. RDEB phenotypes in protein concentration and MFI was calculated by one-tailed Student’s *t*-tests, and significance between age categories and treatments of RDEB samples was calculated by ANOVA with Tukey’s correction.

## Results

### Global metabolic alteration and elevated inflammatory gene expression in RDEB mouse skin

**Figure 1:**
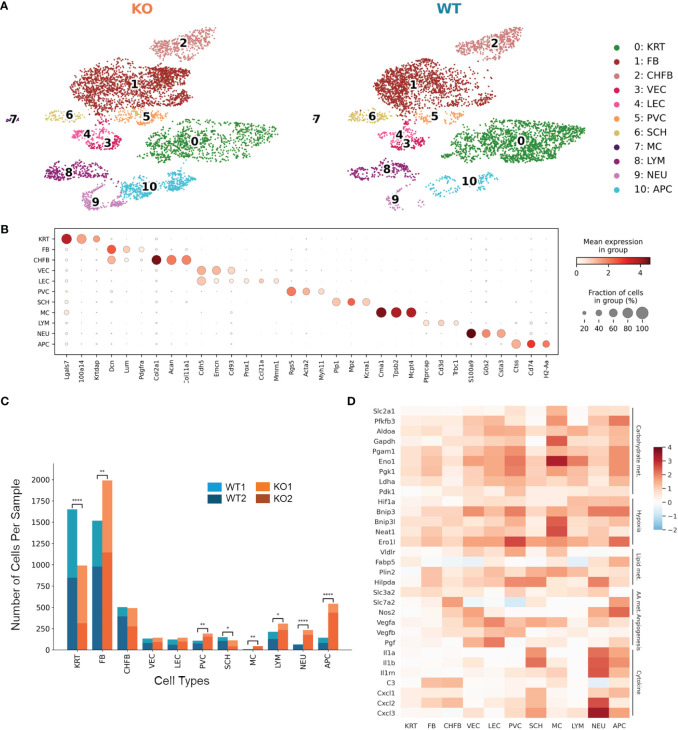
Single-cell RNA-Seq identifies distinct cell populations and transcriptional changes in *Col7a1*^−/−^ mouse skin. **(A)** UMAP plots showing the cell-type composition of the wild-type (WT) and knockout (KO) mouse samples. 0: keratinocytes (KRT), 1: fibroblasts (FB), 2: chondrogenic fibroblasts (CHFB), 3: vascular endothelial cells (VEC), 4: lymphatic endothelial cells (LEC), 5: perivascular cells (PVC), 6: Schwann cells (SCH), 7: mast cells (MC), 8: lymphocytes (LYM), 9: neutrophils (NEU), and 10: antigen-presenting cells (APC). **(B)** Dot plots showing the expression of gene markers by each cell type in the dataset. The color represents the mean expression in each cell type, and the size of the dots represents the percentage of cells within the population that express each marker. **(C)** Barplots showing the number of cells of each cell type in WT and KO mice. ^*^p-value < 0.05; ^**^p-value < 0.01; ^****^p-value < 0.0001; *χ*^2^ contingency test. **(D)** Heat map highlighting upregulated genes in KO samples among all cell types. Color reflects the log-fold difference of gene expression in KO compared to WT. Highlighted genes are grouped together by function: carbohydrate metabolism (Carbohydrate met.), hypoxia, lipid metabolism (Lipid met.), amino acid metabolism (AA met.), angiogenesis, and cytokine and chemokine production (Cytokine).

To focus on an early phase of RDEB disease progression, we isolated single cells from the front paw skin of 11-day-old KO mice (*n* = 2) and WT controls (*n* = 2) for 10x Genomics scRNAseq. We confirmed that both KO mice exhibited typical histological features of RDEB skin, including separation of epidermis and dermis, dermal thickness, infiltration of immune cells, and hyperproliferation of epidermal cells, by sectioning and imaging their hind paws (**Supplementary Figure S1**). Following dimensionality reduction and uniform manifold approximation and projection (UMAP), cells of similar transcriptional profiles were clustered into 11 cell types identified as keratinocytes, fibroblasts, chondrogenic fibroblasts, vascular endothelial cells, lymphatic endothelial cells, perivascular cells, Schwann cells, mast cells, lymphocytes, neutrophils, and antigen-presenting cells (**Figure 1A**). The signature genes defining individual cell types are presented in **Figure 1B** and **Data File S1**. The KO samples contained significantly higher numbers of fibroblasts, perivascular cells, and immune cells, with lower numbers of keratinocytes and Schwann cells than the WT samples (**Figure 1C**).

Differential gene expression between KO and WT samples revealed global as well as cell-type-specific changes (**Data Files S2**, **S3**). Genes classified as hallmark signatures of cancer (35), such as those related to glycolysis, hypoxia-induced autophagy, stress response, fatty acid binding and lipid droplet accumulation, amino acid transport and metabolism, angiogenesis, and proinflammatory cytokines such as *Il1a*, *Il1b*, *C3*, and *Cxcl1*, were upregulated in RDEB samples across all cell types (**Figure 1D**).

Furthermore, several genes whose expression predicts the poor prognosis of many cancer types and represents potential anticancer targets, i.e., *Lgals3*, *Smox*, *Ndrg1*, *Neat1*, and *Rbm39*, were significantly upregulated in most of the KO cell types (**Data File S2**). Overall, these data confirmed the existence of an early proinflammatory environment in RDEB mouse skin. We included a table to highlight notable genes differentially expressed in different cell types (**Table 2**).

**Table 2:** Notable genes in KO samples by major cell types.

Cell type/cluster	Notable gene expression in the KO sample
Keratinocytes/0:KRT	Inflammation and proliferation-related genes*: Krt6a*, *Krt6b*, *Krt16*, *Krt17*
	Epidermal damage reactive genes: *Sprr1a*, *Sprr1b*
	Cytokines and chemokines: *Tslp*, *Il1f9*, *Cxcl16*
Fibroblasts/1:FB	iCAF related genes: *Pdpn*, *Il1rl1*, *C3*, *Saa3*, *Il11*, *Lif*
	Chemoattractant genes: *Ccl2*, *Cxcl1*, *Csf3*
Chondrogenic fibroblasts/2:CHFB	Downregulated collagen genes: *Col2a1*, *Col9a1*, *Col11a1*, *Col11a2*, *Col27a1*
	Inflammatory genes: *Nos2*, *C3*, *Mif*
Vascular endothelial cells/3:VEC Lymphatic endothelial cells/4:LEC Perivascular cells/5:PVC Schwann cells/6:SCH	Angiogenesis: *Vegfa*, *Vegfb*, *Pgf*
Lymphocytes/8:LYM	T-cell costimulatory genes: *Tnfrsf18*, *Tnfrsf9*, *Tnfrsf4*
	T-cell exhaustion genes: *Ctla4*, *Pcdc1*, *Tcf7*, *Foxp3*
Neutrophils/9:NEU Antigen-presenting cells/10:APC	IL-1 signaling genes: *Il1a*, *Il1b*, *Il1rn*, *Nlrp3*
	Immunosuppressive genes: *Cd274*, *Pdcd1lg2*, *Arg1*, *Arg2*
	Chemoattractant genes: *Ccl3*, *Ccl4*, *Ccl5*, *Ccl17*, *Ccl22*, *Cxcl1*, *Cxcl2*, *Cxcl3*, *Csf3*

### Identification of distinct inflammatory and myofibroblast phenotypes in RDEB mouse dermal fibroblasts

**Figure 2:**
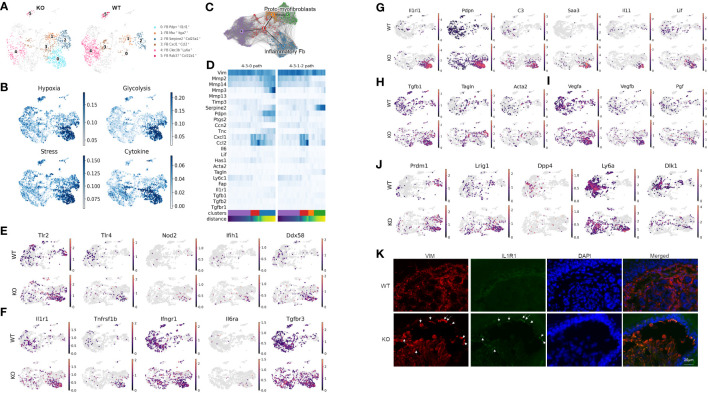
Distinct inflammatory and myofibroblast subsets in RDEB mouse dermal fibroblasts. **(A)** UMAP plots showing the fibroblast subtypes found in wild-type (WT) and knockout (KO) mice. The four subpopulations of interest are highlighted in color, and the rest of the cells are shown in gray. All the subpopulations are described in **Supplementary Figure S3**. **(B)** UMAP plots showing the gene ontology (GO) term scores of hypoxia, glycolysis, stress, and cytokine expression. **(C)** UMAP plot of fibroblast clusters 0, 1, 2, 3, and 4 with partition-based graph abstraction (PAGA) nodes and edges. Two connected nodes are likely to be related in the differentiation trajectory. **(D)** Heatmap of key fibroblast trajectory genes with cells ordered based on trajectory distance. **(E–J)** UMAP plots of genes involved in pattern recognition receptors (PRRs) **(E)**, encoding receptors for key inflammatory cytokines **(F)**, upregulated in cluster 0/*Pdpn*^+^*Il1rl1*^+^ **(G)**, involved in myofibroblast differentiation **(H)**, involved in angiogenesis **(I)**, and defining fibroblast lineages **(J)**. **(K)** Immunohistochemistry staining of vimentin (VIM) and IL-1R1 in the WT and KO paw skin.

Unsupervised clustering within the fibroblast population identified nine subclusters represented by their most differentially expressed gene signatures (**Supplementary Figure S2**, **Data File S4**). **Figure 2A** highlights clusters that either were enriched (subclusters 0, 1, and 2), exhibited different trajectories (subcluster 3), or were significantly less abundant in the KO sample (subclusters 4 and 5). Using gene ontology (GO) enrichment analysis, we identified that subclusters 0, 1, 2, and 3 upregulated genes involved in stress response. More specifically, subcluster 0 and parts of subclusters 1 and 2 expressed genes involved in hypoxia-induced signaling and glycolysis, whereas subclusters 0 and 3 expressed genes related to cytokine and chemokine signaling such as *Ccl2*, *Cxcl1*, and *Csf3* (**Figure 2B**).

While our samples are all from a single time point, the distribution of the subclusters suggests differentiation patterns that could be further elucidated through trajectory analysis. The resulting analysis suggested that subcluster 0 along with subclusters 1 and 2 represent two dynamic processes of inflammatory fibroblast activation and myofibroblast differentiation, respectively (**Figures 2C, D**). Fibroblast subcluster 0, and to a lesser extent subcluster 3, are enriched in gene encoding pattern recognition receptors that sense danger signals and trigger innate immune response, including *Tlr2* and *Tlr4* (Toll-like receptors), *Nod2*, *Ifih1* (MDA5), and *Ddx58* (RIG-1) (**Figure 2E**). These two subclusters were also enriched with the expression of receptors to inflammatory cytokines, i.e., IL-1 receptor (*Il1r1*), TNF receptor 2 (*Tnfrsf1b*), and IL-6 receptor (*Il6ra*) (**Figure 2F**). Meanwhile, the IFN-γ receptor (*Ifngr*) and TGF-β receptor 3 (*Tgfbr3*) were more ubiquitously expressed by all the subclusters of WT and KO fibroblasts (**Figure 2F**). Interestingly, upon transitioning from subcluster 3 into subcluster 0, the expression of *Mmp2*, *Mmp3*, *Mmp13*, *Mmp14*, and *Ptgs2* was significantly upregulated, and the expression of *Timp3* was downregulated (**Figure 2D**), all of which is consistent with the induction of IL-1 signaling (36, 37). Moreover, besides their *Pdpn* and *Il1rl1* signature gene expression, subcluster 0 expressed high levels of *C3*, *Saa3*, *Il11*, and *Lif* (**Figure 2G**), which is reminiscent of the phenotype of IL-1-activated iCAFs. While *Tgfb1* was expressed, the myofibroblast markers, like *Acta2* and *Tagln*, were largely absent in subcluster 0 (**Figure 2H**). In addition, this subcluster is enriched with the angiogenesis and lymphangiogenesis markers *Vegfa*, *Vegfb*, and *Pgf* (**Figure 2I**). We postulate that subcluster trajectory 4–3–0 could be interpreted as a path through which RDEB fibroblasts acquire immune effector functions through activation by inflammatory cytokines such as IL-1 (**Figure 2C**); therefore, we categorized subcluster 0 as inflammatory fibroblasts. In contrast, enhanced expression of *Tagln* and *Serpine2* and downregulation of *Mmp2* in subclusters 1 and 2 suggest that subcluster trajectory 4–3–1–2 may represent a transition toward a myofibroblast phenotype (**Figures 2C, D**) (38–40). Specifically, upregulation of the stress response genes (**Figure 2B**) and low expression of *Ptgs2* (COX2) and *Acta2* (αSMA) (**Figure 2D**) were consistent with the phenotype of subclusters 2 and 1 being proto-myofibroblasts, an intermediate stage of fibroblasts that have responded to mechanical tension and maintain the capacity to differentiate into myofibroblasts upon further stimulation (41). Supportively, a reverse correlation of *Tnc* and *Ccn2* expression was observed between two subcluster trajectories: subcluster trajectory 4–3–0 simultaneously upregulated *Tnc* and downregulated *Ccn2*, while subcluster trajectory 4–3–1–2 appeared to keep the two genes expressed more consistently (**Figure 2D**). This observation is consistent with the reported opposite effects of IL-1α and TGF-β on *Ccn2* and *Tnc* expression (42–44).

scRNAseq analysis also demonstrated that the cells within the KO inflammatory fibroblast subset were mostly positive for *Prdm1* (encoding B-lymphocyte-induced maturation protein 1 (BLIMP1)) and *Lrig1*, and negative for *Dpp4* (CD26) (**Figure 2J**), which suggests that their lineage stems from papillary dermal fibroblasts based on published lineage tracing studies (45). Interestingly, Sca1(*ly6a*)^+^*Dlk1*^+/−^ cells, which characterize the fibroblasts in the lower dermis (hypodermal fibroblasts) (45), were also identified as part of the KO inflammatory fibroblast subset (**Figure 2J**). The recruitment of Sca1^+^ fibroblasts to skin wounds has been demonstrated to be among the initial waves of dermal repair (45). Moreover, Scar1^+^ oral fibroblasts exhibited immunomodulatory functions through the secretion of CCL2 (46). Therefore, the detection of *Ly6a* expression within the KO inflammatory fibroblast subset could represent the activation and recruitment of fibroblasts from the lower to upper dermis in response to dermal–epidermal separations in RDEB skin. Supportively, immunohistochemistry analysis demonstrated that the inflammatory fibroblasts in KO skin, represented by the expression of IL-1R1 and vimentin (VIM), were mainly identified at the dermal–epidermal junction where the blisters occur (**Figure 2K**).

### Recruitment and activation of immune cells in RDEB skin

**Figure 3:**
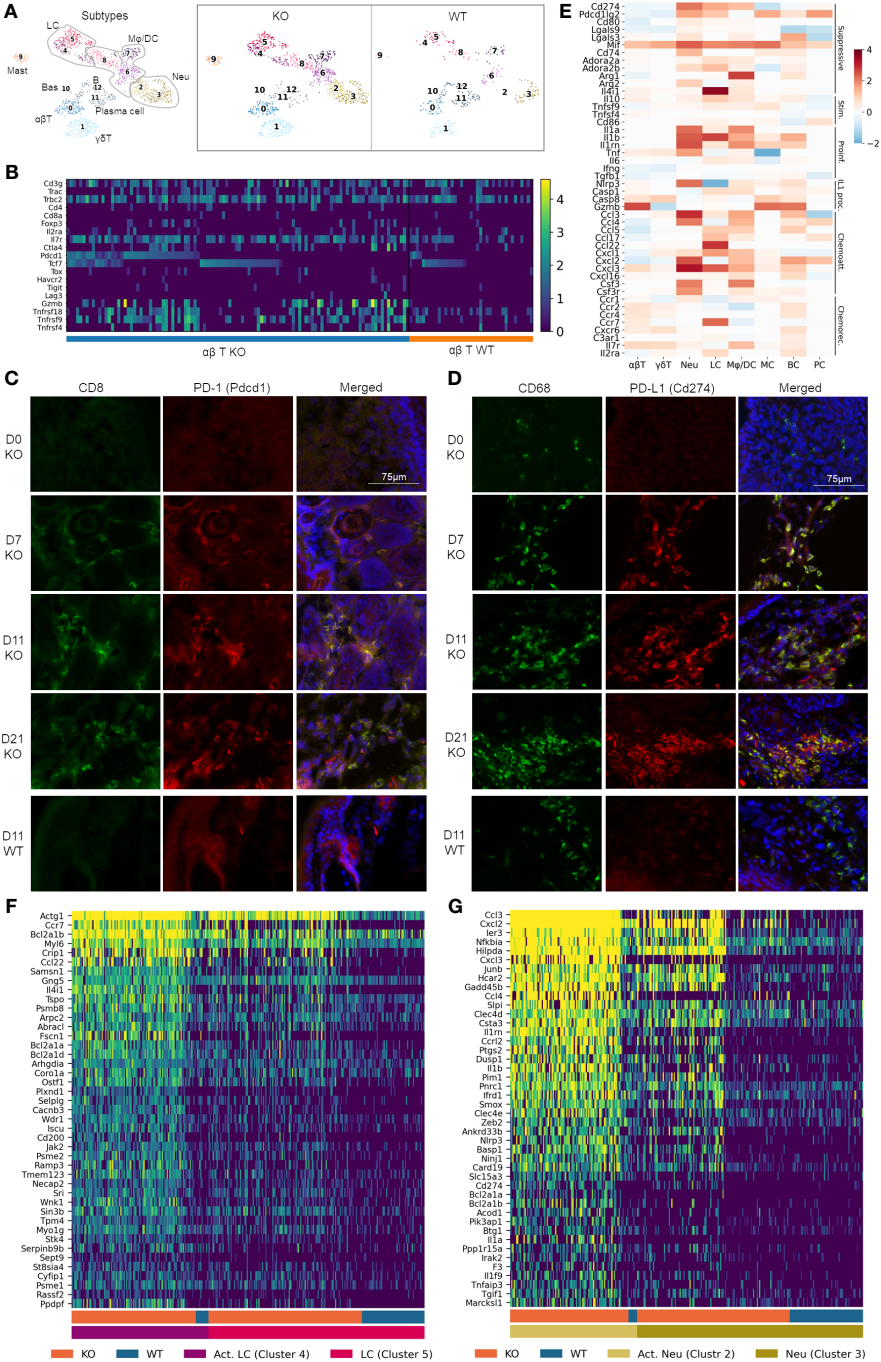
Pervasive recruitment and activation of immune cells in RDEB skin. **(A)** UMAP plots showing the immune cell types in wild-type (WT) and knockout (KO) mice. 0: ɑβ T cells (ɑβT), 1: γδ T cells (γδT), 2 and 3: neutrophils (Neu), 4 and 5: Langerhans cells (LC), 6, 7, and 8: macrophage/dendritic cells (Mφ/DC), 9: mast cells (MC), 10: basophils (Bas), 11: plasma cells (PC), and 12: B cells (BC). **(B)** Heat map highlighting ɑβ T-cell gene expression at a single-cell level. Cells were grouped by experimental condition (KO vs. WT) and ordered along the *x*-axis by expression of *Pdcd1* and *Tcf7*. **(C, D)** Accumulation of CD8^+^PD-1^+^ T cells and CD68^+^PD-L1^+^ macrophages in immunohistochemically stained KO paw skin over a 3-week time course. Images were representative of three biological samples per group. Time points for staining are 0 (D0), 7 (D7), 11 (D11), and 21 (D21) days after birth. The staining in the 11-day-old wild-type (D11 WT) paw skin was representative of all WT time points. CD8/PD-1 and CD68/PD-L1 staining in C7^hypo^ mouse skin is included in **Supplementary Figure S4**. **(E)** Heat map on select genes, grouped together by function: immune suppressive (Suppressive), immune stimulatory (Stim.), proinflammatory (Proinf.), IL-1 processing (IL1 proc.), chemoattractants (Chemoatt.), and chemokine receptors (Chemorec.). Color reflects the log-fold difference of gene expression in KO compared to WT. **(F, G)** Heatmaps highlighting Langerhans cell and neutrophil gene expression at a single cell level. Cells were grouped together by WT and KO and activated (Act. LC/Act. Neu) and nonactivated (LC/Neu) Langerhans cells/neutrophils.

Unsupervised clustering among immune cells identified 12 immune cell types/subtypes (**Figure 3A**; **Data File S5**). Clusters 0 and 1 were identified as αβ and γδ T cells, based on their expression of *Trac* and *Trbc* and *Tcrg-c1* and *Trdv4*, respectively. Clusters 2 and 3 expressed neutrophil markers *S100a8*, *S100a9*, and *Ngp* and thus were identified as neutrophils, of which cluster 2 represented an activated neutrophil subpopulation unique in the KO sample. Similarly, clusters 4 and 5 together were identified as Langerhans cells based on their expression of *Cd74* and *Cd207*, *with* cluster 4 representing an activated Langerhans cell subpopulation. The number of cells within clusters 6, 7, and 8 was significantly higher in the KO samples than in the WT samples, and these clusters were largely positive for the expression of *H2-Ab1*, *Adgre1*, *Cd68*, *Fcrg1*, and *Itgam*. For the scope of this manuscript, we grouped these clusters as macrophages/dendritic cells (Mφ/DC).

The αβ T cells contained conventional *Cd4*^+^*Foxp3*^−^ T helper cells, *Cd8*^+^ cytotoxic T cells, and *Foxp3*^+^ T regulatory (Treg) cells (**Figure 3B**). While *Cd8* transcript levels were low in our data analysis, immunohistochemical (IHC) analysis demonstrated an abundant amount of CD8^+^ cells in the dermis of KO paw skin (**Figure 3C**). Meanwhile, CD8^+^ cells were absent in the WT paw skin (**Figure 3C**), which was consistent with the reported immunological features of murine paw skin (47). Therefore, we postulated that the low *Cd8* detection in our KO dataset could be due to the limited sequencing depth of the scRNAseq technique not fully representing the transcriptional complexity of T cells. It may also be due to an incoherence between mRNA and protein expression (48). Nevertheless, our data demonstrated that the KO αβ T cells exhibited upregulated expression of both inhibitory (e.g., *Pdcd1/*PD-1, *Ctla4*) and costimulatory (e.g., *Tnfrsf9*/4-1-BB, *Tnfrsf18*/GITR, and *Tnfrsf4*/OX40) genes (**Figure 3B**), a phenomenon that has been documented in T cells within the tumor microenvironment (12, 49). Here, IHC analysis demonstrated that CD8^+^ T cells were PD-1^+^ when recruited to the KO mouse skin a week after birth (**Figure 3C**), suggesting that these cells were effector cells of adaptive immunity. The number of T cells and their expression of PD-1 remained elevated in the KO skin for the following 2 weeks (**Figure 3C**). Persistent PD-1 expression was also observed in T cells in the skin of C7^hypo^ mice that survived long-term and exhibited mitten deformity (**Supplementary Figure S3A**) consistent with the previous study (30) and suggestive of a process of T-cell exhaustion in the chronic inflammatory environment of RDEB skin. Interestingly, scRNAseq analysis revealed that *Pdcd1*^+^ T cells in KO samples can be separated into *Tcf7*-positive and negative subpopulations (**Figure 3B**). *Tcf7* encodes T-cell factor-1 (TCF-1) that marks either naïve or central memory stem-like precursor CD8^+^ T cells with self-renewal capacity (50). Importantly, *Pdcd1*^high^*Tcf7*^+^ T cells with low expression of coinhibitory receptors such as *Havcr2* (TIM-3), *Tigit*, and *Lag3* in the KO sample (**Figure 3B**) were consistent with the phenotypes of progenitor-exhausted T cells that are long-lived, have stem cell-like properties, and respond to immune checkpoint blockade therapy (51). In contrast, the expression of *Tox*, which plays a crucial role in the generation and maintenance of an exhausted T-cell phenotype (52), and other coinhibitory receptors *Havcr2*, *Tigit*, and granzyme B (*Gzmb*), were more associated with the *Pdcd1*^+^*Tcf7*^−^ T cells in the KO samples (**Figure 3B**).

Correlated with increased expression of *Pdcd1* in KO T cells, elevated expression of *Cd274* (PD-L1) and/or *Pdcd1lg2* (PD-L2) was observed in Langerhans cells, neutrophils, and macrophages/dendritic cells (**Figures 3D, E**). Interestingly, although CD68^+^ cells were identified in the dermis of newborn KO paw skin, they had no expression of PD-L1 (**Figure 3D**). In accordance with the timing of PD-1^+^ T-cell recruitment, PD-L1 was identified in CD68^+^ cells in the paw skin of 7-day-old and older KO mice, suggestive of the activation of the PD-1/PD-L1 axis in RDEB immune cells (**Figure 3D**). PD-L1 expression was also observed in the CD68^+^ cells in C7^hypo^ mice with mitten deformity (**Supplementary Figure S3B**) but was not identified in the WT CD68^+^ cells at any time points (represented by the d11-timepoint in **Figure 3D**). In addition, although the expression of costimulatory molecules such as *Tnfsf9* (4-1BBL), *Tnfsf4* (OX40L), and *CD86* was increased in some KO immune cell types, the extent of their upregulation was overall less than the elevation of immune suppressive molecules such as *Lgals9*, *Lgals3*, *Mif*, *CD74*, *Adora2a*, *Adora2b*, *Arg1*, *Arg2*, and *Il4i1* (**Figure 3E**).

Significant upregulation of *Il1a* (IL-1α), *Il1b* (IL-1β), and their antagonist *Il1rn* (IL-1ra) was identified in multiple immune cell types in the KO sample (**Figure 3E**), further supporting the subcluster trajectory of inflammatory fibroblast activation. Moreover, genes encoding inflammasome sensor proteins (Nlrp3) and proteolytic enzymes (*Casp1*, *Casp8*, and *Gzmb*) were significantly upregulated in multiple immune cell types in the KO sample. These enzymes are essential not only for processing pro-IL-1β into an active form but also for pro-IL-1α to further increase its bioactivity, even though IL-1α is active in its full-length form (53). Upregulation of other cytokines, such as *Tnf and Il6*, was also observed in multiple immune cell types in the KO sample (**Figure 3E**). Moreover, chemoattractant proteins were highly expressed by Langerhans cells, macrophages/dendritic cells, neutrophils, and B cells in the KO sample, while chemokine receptors were upregulated in T cells and B cells (**Figure 3E**), suggesting their recruitment by these chemotactic axes.

A heatmap of single cells within the Langerhans cell population of WT and KO samples demonstrated that the novel cluster 4 in the KO sample exhibited significant upregulation of antiapoptosis genes, such as *Bcl2a1a*, and genes involved in actin filament binding, lamellipodium assembly, podosome formation, and cell projection and adhesion, such as *Abracl*, *Wdr1*, and *Fscn1* (**Figure 3F**). Together with the significant upregulation of *Ccr7*, induced only at later stages of Langerhans cell maturation and endowing migratory capacity (54), our results suggested that this novel cluster could represent mobilized Langerhans cells migrating to lymph nodes in RDEB mice. The migration of Langerhans cells is an essential mechanism for the induction of Tregs and can be induced by IL-1 or TNF (55).

The heatmap of single cells within clusters 2 and 3 also revealed neutrophil activation in KO samples, exhibited by the upregulation of genes involved in neutrophil chemotaxis and chemokine-mediated signaling, IL1 signaling, TNF signaling, NF-кB signaling, C-type lectin receptor signaling, Toll- and NOD-like receptor signaling, integral components of membrane composition, as well as antiapoptotic regulation gene activation (**Figure 3G**). Moreover, this activation appears to have occurred with a gradual, three-stage progression. One part of cluster 3 contains lower gene expression and is present in both WT and KO samples. The other part of cluster 3 contains a slightly elevated gene expression and is uniquely expressed in the KO sample. In contrast, cluster 2 is almost exclusively found in the KO sample and has the highest expression of these genes (**Figure 3G**).

Overall, these data suggested pervasive recruitment and activation of immune cells in RDEB skin that supported the induction of inflammatory fibroblast activation trajectory in the dermal microenvironment.

### Dysfunctional epidermal barrier formation and keratinocyte-immune cell crosstalk in RDEB

**Figure 4:**
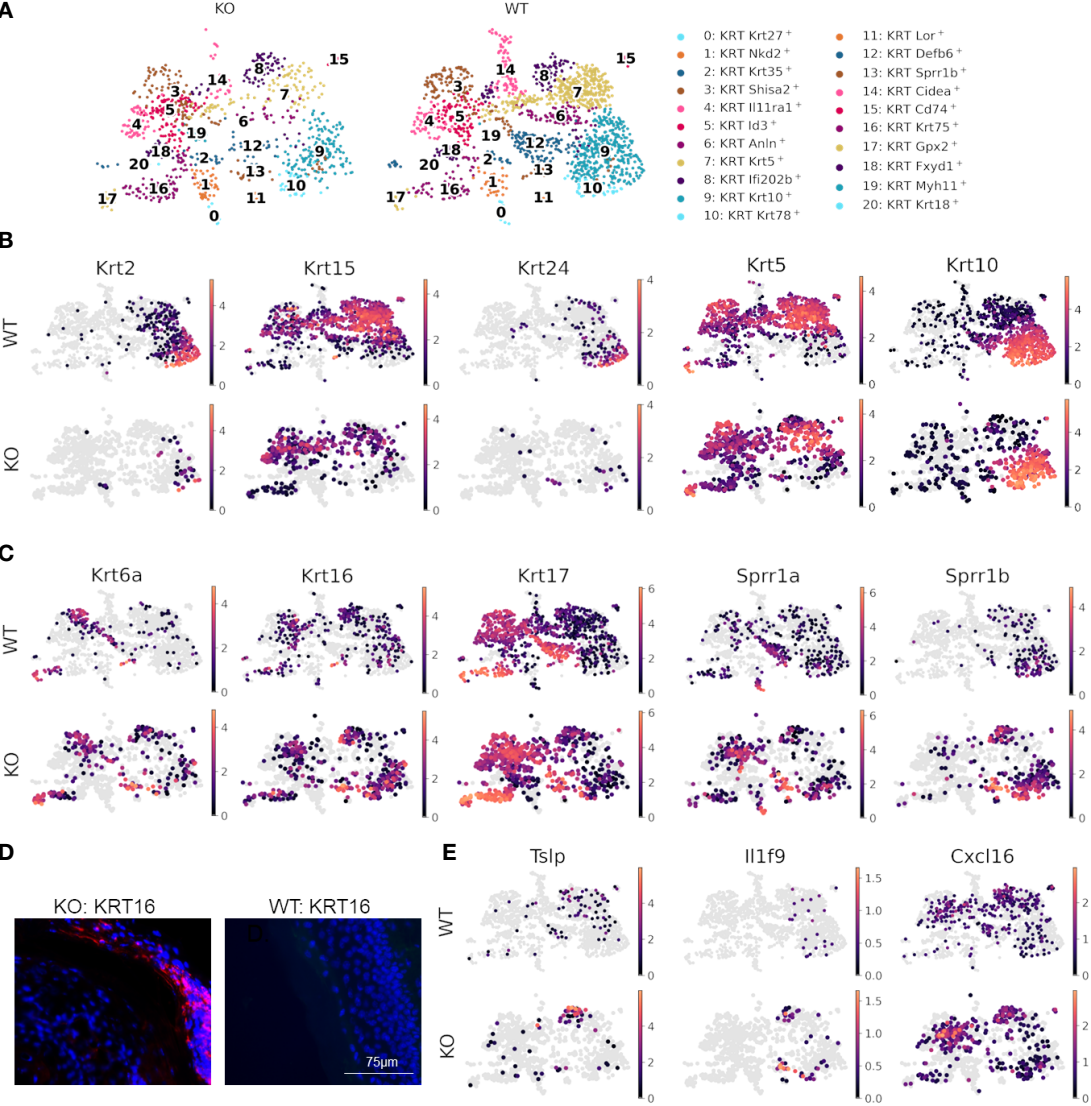
Dysfunctional epidermal barrier formation and keratinocyte-immune cell crosstalk in RDEB. **(A)** UMAP plots showing keratinocyte subtypes in wild-type (WT) and knockout (KO) mouse samples. **(B)** Significantly downregulated and **(C)** upregulated genes in KO keratinocytes shown by UMAP plots of their expression in WT and KO keratinocytes. Images were representative of three biological samples per group. **(D)** Representative KRT16 immunohistochemical staining in the epidermis of KO and WT mice. **(E)** UMAPs highlighting inflammatory genes upregulated in keratinocytes of KO mice.

Since skin compartments are tightly interconnected, we next explored signaling at the epidermal level. Differential expression analysis between WT and KO keratinocytes revealed that multiple keratin genes, *Krt2*, *Krt5*, *Krt10*, *Krt15*, and *Krt24*, were significantly downregulated, and *Krt6a*, *Krt6b*, *Krt16*, *Krt17*, *Sprr1a*, and *Sprr1b* were significantly upregulated in KO keratinocytes as compared to the WT (**Supplementary Tables S2**, **S3**). Subclustering and UMAP analysis of keratinocytes (**Figures 4A–C**) demonstrated that most of the differential expression occurred within interfollicular epidermal basal cells (clusters 7 and 8), suprabasal cells (clusters 9 and 10), and germinative layer cells (cluster 5), based on the expression patterns of each subcluster (**Data File S6**) (56). The imbalanced expression of *Krt and Sprr* genes in RDEB mouse keratinocytes was consistent with a previous transcriptome analysis of RDEB patients’ skin (57). Importantly, the downregulation of *Krt*2/15 and upregulation of *Krt*6/16/17 have been associated with inflammation and the proliferative stage of keratinocyte differentiation under pathological conditions such as psoriasis (58–60). Supportively, strong KRT16 immunostaining was observed in the suprabasal epidermal layer of KO skin, particularly in the area with hyperkeratosis (**Figure 4D**). The KO keratinocytes also had upregulated expression of *Sprr1a* and *Sprr1b* (**Figure 4C**), suggesting their role in protecting against the damaged skin barrier in RDEB. However, *Sprr1b* has also been demonstrated to be overexpressed in multiple types of cancer with poor prognosis (61, 62).

Importantly, the epidermal basal cells in cluster 8 of KO samples were also enriched with the expression of inflammatory cytokines and chemokines, including *Tslp*, *Il1f9* (IL-36γ), and *Cxcl16* (**Figure 4E**), all of which have been demonstrated to contribute to the pathogenesis of psoriasis (63–65). Thymic stromal lymphopoietin (TSLP) is well known for its association with type 2 immunity at the barrier surface, allergic conditions, chronic inflammatory diseases, and cancer (66). It has been demonstrated that following the binding of TSLP to its receptor TSLPR on immune cells, the TSLP-TSLPR complex recruits IL-7R with high affinity to activate TSLP signaling (67). This event thus sequesters IL-7R from IL-7R signaling and may further influence IL-2R signaling, as IL-7R and IL-2R share a common γ-chain for their respective activation (68). Therefore, the upregulation of *Tslp* may be one of the mechanisms underlying the crosstalk between keratinocytes and immune cells in the RDEB skin. Importantly, not only was *Tslp* upregulated in keratinocytes, but *Il7r* was also significantly upregulated in both T cells and macrophages/dendritic cells of the KO sample (**Figures 4B, E**). Moreover, recent studies demonstrated that following inflammatory stimulation, monocytes and macrophages were more likely to produce soluble IL-7R (sIL7R), a decoy for IL-7R signaling, than full-length IL-7R, which is normally produced by T cells (69). Therefore, there appeared to be multiple levels of immunomodulation in the KO sample, which warrants further investigation.

### Multiple waves of inflammatory responses in RDEB mice

In line with specific immune regulation within the major cell types described above, the ingenuity pathway core analysis on globally differential gene expression demonstrated that immune responses, centered on IL-1 and regulated by other cytokines, constitute a major part of the upregulated signaling network in RDEB samples (**Supplementary Figure S4**). Therefore, we next profiled key cytokines in the skin lysate of KO mice and WT controls. Specimens from C7^hypo^ mice were included with KO as an additional validation of our findings and to increase the robustness of our data analysis.

**Figure 5:**
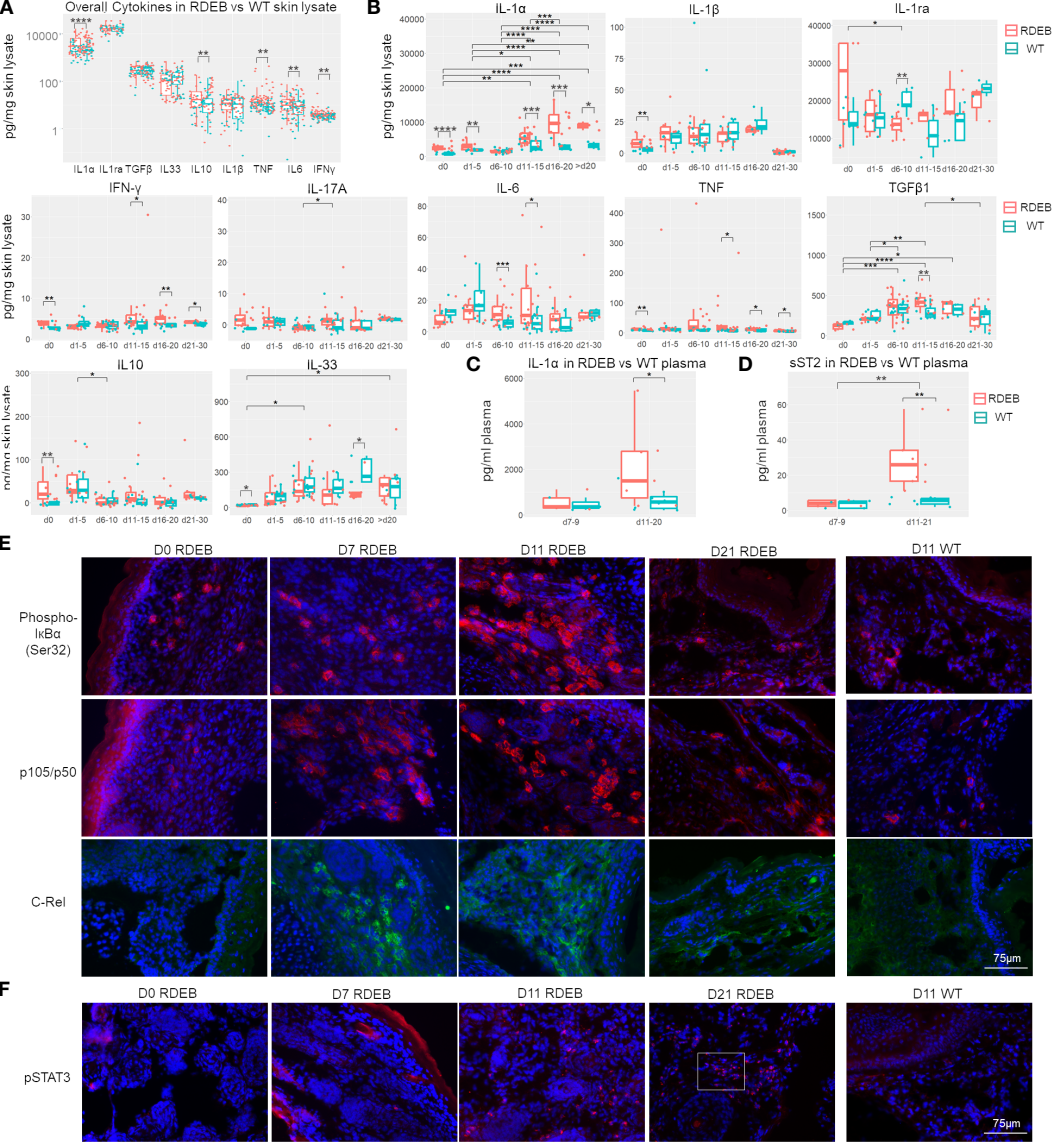
RDEB mice exhibit distinct waves of inflammation after birth. **(A, B)** Quantitation of cytokine concentrations in paw skin lysates of RDEB and wild-type (WT) mice. Results show box and scatter plots of RDEB (*n* = 46–73) and WT (*n* = 36–48). ^*^p-value < 0.05; ^**^p-value < 0.01; ^***^p-value < 0.001; ^****^p-value < 0.0001. Data between age groups of RDEB samples were analyzed by ANOVA with Tukey’s correction, and data between RDEB and WT samples within each age group were analyzed by unpaired Student’s *t*-test. **(A)** Comparison of cytokine log concentrations in RDEB and WT paw skin. **(B)** Individual cytokine expression differential between RDEB and WT mouse paws grouped by age (d0 = 0 days old, d1–d5 = 1 to 5 days old … d21–d30 = 21 to 30 days old). Representative cytokine levels spread by age are presented in **Figure S7**. **(C, D)** Quantitation of IL-1α **(C)** and sST2 **(D)** concentrations in the plasma of RDEB and WT mice. Results show box and scatter plots of RDEB (*n* = 10–13) and WT (*n* = 11–13) grouped by age. **(E, F)** Immunohistochemistry analysis of NF-κB (phospho-IκBɑ (Ser32), p105/p50, and C-Rel) and JAK/STAT (pSTAT3) signaling in RDEB and WT paw skin sections. A magnified inset image in **(F)** is shown in **Supplementary Figure S8C**. Images were representative of three biological samples per group. Scale bar: 75 µm.

Our results suggest that IL-1α, IL-10, TNF, IL-6, and IFN-γ were significantly elevated overall in the RDEB skin lysate compared the WT control (**Figure 5A**). The mean concentration of IL-1ra was high, but it was not significantly different between RDEB and WT mice. In addition, although scRNAseq showed a significantly higher expression of *Il1b* in RDEB samples, quantitation of IL-1β through two independent methods revealed similarly low levels in both groups.

Importantly, dynamic profiling of individual cytokines suggests that there are likely multiple waves of immune responses in RDEB skin from birth to early adulthood (**Figure 5B**; **Supplementary Figure S7**). At birth, RDEB skin contained significantly higher levels of both proinflammatory (IL-1α, IL-1β, TNF, IFN-γ, and IL-17A) and anti-inflammatory (IL-10) cytokines than the WT skin (**Figure 5B**). Among these, IL-1α exhibited the highest magnitude of elevation, with its concentration > 2.8-fold higher in the RDEB than WT skin. Meanwhile, the level of IL-33 was significantly lower in the RDEB skin than in the WT skin at birth. At postnatal days 1–5, IL-1α was still significantly elevated in the RDEB samples, while the other cytokines had lost the significant difference between the RDEB and WT samples. Interestingly, at around a week of age (d6–d10), the level of IL-1α came down in RDEB skin. The decreased level of IL-1α was accompanied by a significant decrease in IL-1ra and a significant increase in IL-6. The level of TNF was also much higher in the RDEB skin than in the WT, although it was not statistically significant due to the high variability caused by spikes in TNF concentrations in some KO samples. Importantly, from postnatal day 11 to day 20, the level of IL-1α was again significantly upregulated in RDEB, not only compared to the WT but also to the RDEB lysate at earlier time points (**Figure 5B**; **Supplementary Figure S5**). Moreover, the concentrations of IFN-γ, IL-6, TNF, and TGF-β1 were all elevated. In contrast, the level of IL-33 significantly decreased. The levels of all the proinflammatory cytokines appeared to diminish when the RDEB mice reached young adulthood; however, the concentrations of IL-1α, TNF, and IFN-γ were still significantly higher in the RDEB than in the WT skin.

The levels of IL-1α in the plasma also reflected the dynamics of IL-1α in the RDEB skin lysate: they were not significantly different between the RDEB and WT mice at around 1-week of age; however, were significantly elevated at later days (**Figure 5C**). Of note, there was no significant difference between the KO and C7^hypo^ mice in the dynamics of all the measured cytokines except IL-1α (**Supplementary Figure S6A**). While the C7^hypo^ skin still had significantly higher levels of IL-1α than the WT skin, its level was significantly lower than the IL-1α in KO skin, suggesting that the complete lack of C7 resulted in a higher magnitude of IL-1α production (**Supplementary Figure S6A**). Importantly, we also detected suppression of tumorigenicity 2 (ST2) in the plasma of RDEB mice, resembling the dynamics of IL-1α (**Figure 5D**). ST2 is a member of the IL-1-receptor superfamily encoded by *Il1rl1*, a gene that was upregulated predominantly in the inflammatory fibroblast subset (cluster 0) in the KO samples (**Figure 2**). There are two forms of ST2, a membrane-bound form (STL) expressed primarily in hematopoietic cells and mediating signaling upon IL-33 binding and a soluble form (sST2) largely inducible in fibroblasts, serving as a decoy receptor for IL-33/ST2L and a serological biomarker for fibrosis (70). The observed decrease of IL-33 in RDEB skin lysate and increase of sST2 in RDEB plasma were thus suggestive of a downregulation of the IL-33/ST2L signaling axis in RDEB. However, the full extent to which sST2 might contribute to the pathological condition of RDEB remains to be determined.

As expected from the function of IL-1α and/or TNF on inducing NF-κB signaling, phosphorylated Iκ-Bα (pIκBα) was identified in the newborn RDEB skin, suggesting that NF-κB signaling is initiated before or at birth (**Figure 5E**). Upregulation of this signaling became apparent at a week of age, was further enhanced at 11 days of age, determined by the significant elevation in the levels of NF-κB subunits, p50/p105 and C-Rel and nuclear localization of p50, and appeared to be lower in the following timepoints (**Figures 5E**; **Supplementary Figure S6B**). Interestingly, phosphorylated STAT3 (pSTAT3) was absent in the newborn KO skin, sporadically detected at a week of age, became significantly positive at 11 days, and stayed high at 3 weeks of age (**Figure 5F**; **Supplementary Figure S6C**), suggesting that JAK/STAT signaling was activated later than NF-κB signaling. Moreover, p50/p105 and pSTAT3 were positive in the skin of 3-month-old C7^hypo^ mice (**Supplementary Figure S6D**), suggesting the persistent activation of both signaling pathways. In contrast, all these factors were at a low level or undetectable in the WT skin (**Figures 5E, F**). The results were congruent with the later peak of IL-6 and IFN-γ, two potent activators of JAK/STAT signaling in the RDEB skin, and with the reported IL-1-induced signaling cascade that leads to JAK/STAT activation in iCAFs (15).

**Figure 6:**
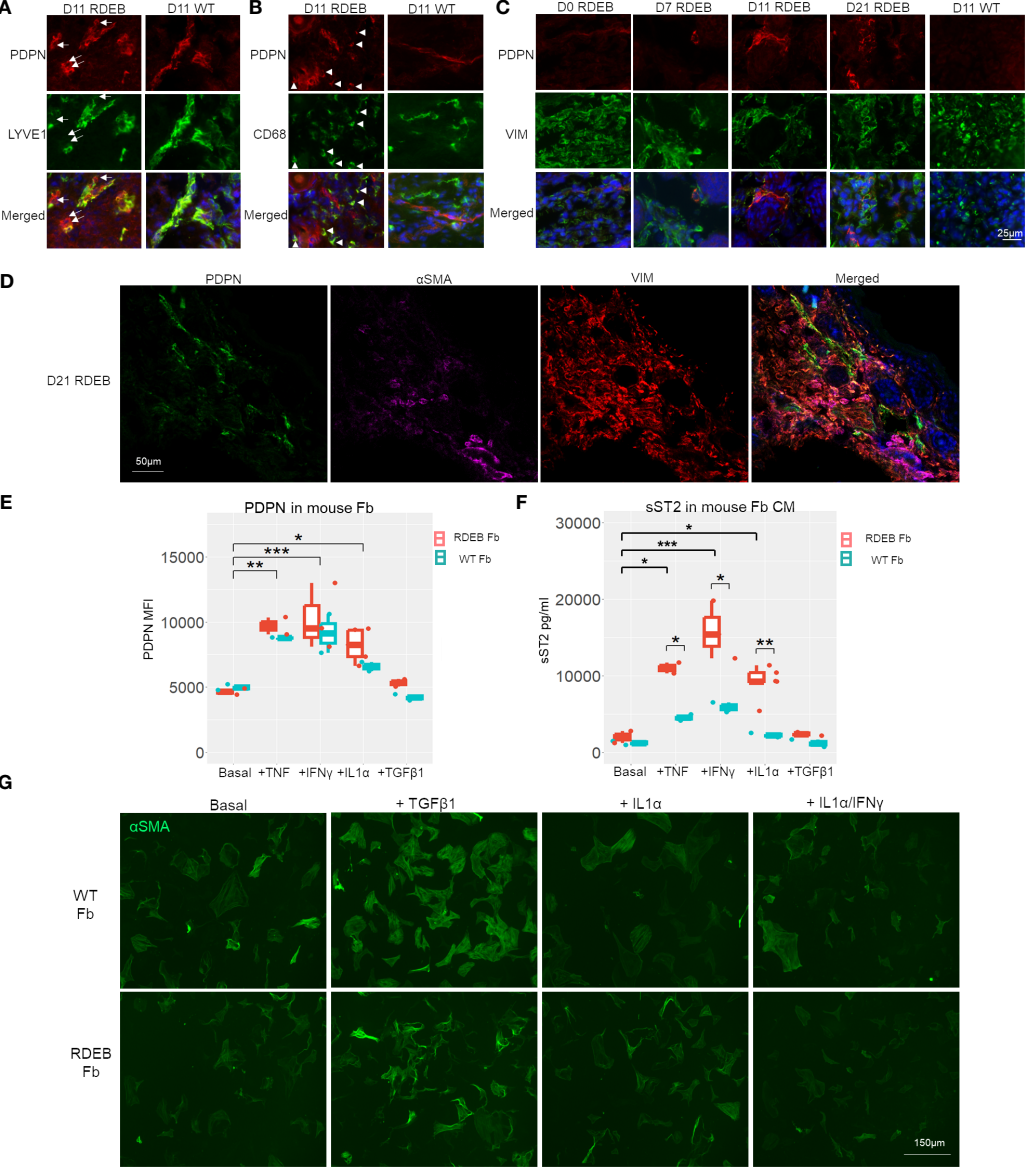
PDPN expression in wild-type (WT) and RDEB mice and *in vitro* inflammatory responses of WT and RDEB mouse-derived fibroblasts. **(A–C)** Immunohistochemistry (IHC) staining highlighting the colocalization of PDPN with vascular cells (lymphatic vessel endothelial hyaluronan receptor 1 (LYVE1)), macrophage/dendritic cells (CD68), and fibroblasts (vimentin (VIM)) in the skin of RDEB and WT mouse paws. Images were representative of three biological samples per group. White arrows in **(A)** identify PDPN^+^LYVE1^−^ cells, and white arrowheads in **(B)** identify PDPN^+^CD68^+^ cells in RDEB paw skin. In the WT paw skin, PDPN^+^ cells were mainly colocalized with LYVE^+^ vascular cells. **(D)** IHC analysis of PDPN, αSMA, and VIM staining in D21 RDEB mouse skin. **(E, F)** *In vitro* responses of RDEB and WT mouse fibroblasts (Fb) under basal conditions and following the treatment with TNF, IFN-γ, IL-1α, and TGF-β1, respectively. *p-value < 0.05; **p-value < 0.01; ***p-value < 0.001. **(E)** Median fluorescence intensity (MFI) of PDPN staining measured by flow cytometry and **(F)** ST2 in the fibroblast-conditioned medium (CM) quantitated by ELISA analysis. Data between treatments of fibroblasts were analyzed by ANOVA with Tukey’s correction, and data between RDEB fibroblasts and normal controls within each treatment were analyzed by paired Student’s *t*-test. **(G)** Immunocytochemistry staining of αSMA in WT and RDEB fibroblasts under basal condition and following treatment with TGF-β1, IL-1α, and IL-1α/IFN-γ.

We next evaluated the dynamics of inflammatory fibroblasts in RDEB mouse skin based on the expression of PDPN. PDPN is a mucin-type transmembrane protein that is normally expressed on lymphatic endothelial cells and fibroblastic reticular cells of lymphoid organs. PDPN is also utilized as a biomarker for CAFs, and its overexpression in CAFs, epithelial tumor cells, and inflammatory macrophages is associated with worse outcomes in many cancers (71). In WT mouse skin, PDPN expression was mainly associated with the vascular cells (LYVE^+^) (**Figure 6A**), whereas in RDEB mouse skin, in addition to its positive staining on the vascular cells, PDPN was associated with both fibroblasts (VIM^+^) and CD68^+^ immune cells (**Figures 6A–C**). Moreover, in line with the dynamics of JAK/STAT signaling, PDPN expression on fibroblasts was rare in the skin of RDEB mice less than a week of age and was substantially increased in the skin of older RDEB mice (**Figure 6C**). Importantly, PDPN^+^ fibroblasts were distinct from the αSMA^+^ myofibroblasts (**Figure 6D**), consistent with the scRNAseq analysis that suggested distinct trajectories of inflammatory fibroblast and myofibroblast differentiation in RDEB mouse skin.

### Inflammatory responses of RDEB mouse and human patient-derived fibroblasts to proinflammatory cytokines *in vitro*

It has been well established that inflammatory cytokines such as TNF, IL-1, and IL-17 are potent stimulators of fibroblast activation in inflammatory diseases (19, 72). We, therefore, asked whether stimulation of fibroblasts with the cytokines that were prominently elevated in RDEB mouse skin lysates would lead to upregulations of inflammatory fibroblast signature genes identified in our scRNAseq analysis, i.e., expression of PDPN and secretion of ST2, and whether there is any differential response between RDEB-derived and normal fibroblasts.

Here, we isolated fibroblasts from RDEB mice and WT controls at a week of age and analyzed their responses to mouse IL-1α, TNF, IFN-γ, and TGF-β individually following 24-h starvation in a serum-free (basal) medium. We demonstrated that PDPN expression on the cell surface and ST2 release in the conditioned medium (CM) were detected at similar levels in both RDEB and WT mouse fibroblasts under starved conditions and could be significantly enhanced under various stimulus conditions, confirming that they are fibroblast inflammatory responders (**Figures 6E, F**). TNF, IL-1α, and IFN-γ treatments all individually significantly enhanced PDPN expression and ST2 secretion in both RDEB and WT mouse fibroblasts, while TGF-β1 had no stimulatory effects on their expression (**Figures 6E, F**). Conversely, the addition of TGF-β1, but not IL-1α or IL-1α/IFN-γ, increased the fibroblast expression of αSMA, consistent with the effect of TGF-β1 on myofibroblast differentiation (**Figure 6G**). There was a trend of higher PDPN expression in RDEB fibroblasts compared to WT mouse fibroblasts under stimulatory conditions; however, it was not statistically significant (**Figure 6E**). ST2 was secreted at a significantly higher amount by RDEB mouse fibroblasts than WT following stimulation with TNF, IL-1α, and IFN-γ, respectively (**Figure 6F**).

**Figure 7:**
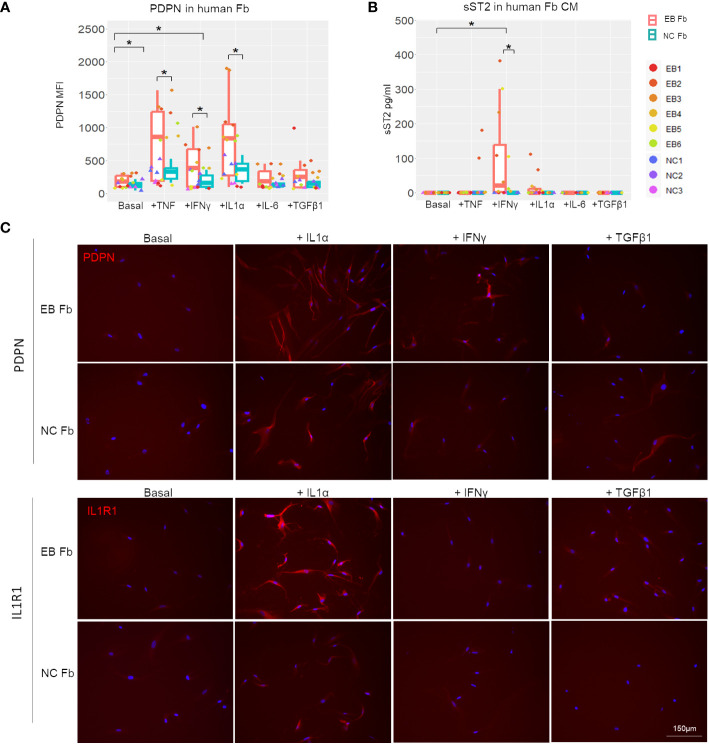
*In vitro* inflammatory responses of RDEB patient-derived and normal control fibroblasts. RDEB patient-derived (EB Fb) and normal control (NC Fb) fibroblasts were cultured for 24 h with cytokines (TNF: 100 ng/mL, IFN-γ: 100 ng/mL, IL-1α: 4 ng/mL, IL-6: 2 ng/mL, and TGF-β1: 5 ng/mL) in basal media. **(A)** Flow cytometry analysis of PDPN expression measured by median fluorescence intensity (MFI). Results show box and scatter plots of RDEB patients (*n* = 6) and normal controls (*n* = 3) with two replicates each, grouped by cytokine treatment. **(B)** ST2 concentrations measured in conditioned media (CM) by ELISA. Results show box and scatter plots of RDEB patients (*n* = 6) and normal controls (*n* = 3) with one to two replicates each, grouped by cytokine treatment. ^*^p-value < 0.05. Data between treatments of fibroblasts were analyzed by ANOVA with Tukey’s correction, and data between RDEB fibroblasts and normal controls within each treatment were analyzed by paired Student’s *t*-test. See **Supplementary Figure S9** for overlaid flow cytometry plots of PDPN and membrane-bound ST2 (ST2L) expression of a representative RDEB patient-derived fibroblast at a basal level and following the treatment of cytokines. **(C)** Immunocytochemistry staining of PDPN and IL-1R1 in EB Fb and NC Fb under basal conditions and following treatment with IL-1α, IFN-γ, and TGF-β1, respectively. Scale bar: 150 µm.

We also analyzed RDEB human patient-derived fibroblasts (*n* = 6; **Table 1**) and normal control fibroblasts (*n* = 3) under basal conditions and following stimulation with recombinant human IL-1α, TNF, IFN-γ, IL-6, or TGF-β (**Figure 7**). A notable discrepancy between human and mouse fibroblasts is that under basal conditions, the expression of PDPN and ST2 was almost undetectable in human fibroblasts, whereas mouse fibroblasts expressed a detectable amount of both under basal conditions. However, consistent with the postulated trajectory of inflammatory fibroblast stimulation, they are significantly upregulated in response to inflammatory cytokine stimulation, similarly in both mouse and human fibroblasts. Moreover, TNF, IL-1α, or IFN-γ enhanced PDPN expression to a significantly higher level in RDEB patient-derived fibroblasts compared to normal controls (**Figures 7A, C**; **Supplementary Figure S5**). As for ST2, normal control fibroblasts did not produce ST2 under any condition (**Figure 7B**). While IL-1α stimulated certain RDEB patient-derived fibroblasts to secrete ST2, IFN-γ was the most potent ST2 stimulator among all RDEB patient-derived fibroblasts (**Figure 7B**). Membrane-bound ST2 (STL) was not detectable within all the stimulatory conditions (**Supplementary Figure S7B**), confirming the production of sST2 from the *IL1RL1* gene in RDEB patient-derived fibroblasts. In addition, immunocytochemistry staining demonstrated that expression of the receptor to IL-1α, i.e., IL-1R1, can be induced specifically by IL-1α, and the level of its induction was more robust in RDEB patient-derived fibroblasts than normal controls (**Figure 7C**).

## Discussion

Our scRNAseq analyses have suggested many irregularities in the RDEB mouse dermal microenvironment, including altered metabolism, enhanced angiogenesis, dysfunctional epidermal barrier, infiltration and activation of immune cell populations, and inflammatory fibroblast activation. Together with the dynamics of proinflammatory cytokines in RDEB mice and the responses of RDEB patient-derived fibroblasts to cytokine stimulation, our data underscore the essential role of inflammation in RDEB pathophysiology and suggest a pathway to the development of a protumor microenvironment that may underlie the early onset and aggressive nature of RDEB-associated cSCC.

Cross-tissue examination of fibroblasts by scRNAseq analysis has empowered high-resolution detection of diverse subtypes across a spectrum of inflammatory and fibrotic diseases and in various cancers. Intriguingly, the inflammatory fibroblast subset in RDEB mice shared important features, i.e., expression of PDPN, CC, and CXC chemokines and cytokines, with the synovial fibroblast subset found in patients with rheumatoid arthritis (73) or the ileum fibroblast subset found in patients with inflammatory bowel disease (74). Many genes that were significantly upregulated in the RDEB inflammatory fibroblast subset, e.g., *Il11*, *Lif*, *Vegf*, *Saa3*, *Tnc*, and *C3*, are also the signature genes of iCAFs identified in many cancers. These factors have been demonstrated to enhance tumor cell growth and metastasis; thus, iCAFs have emerged as a novel target in anticancer immunotherapy (75). In parallel, inflammatory disease therapies are being developed to target inflammatory fibroblasts to prevent their activation, neutralize effector molecules, or deplete them together (72). In the current study, this inflammatory fibroblast subset was identified by scRNAseq in RDEB mice at an early phase of pathological progression without malignant transformation. The expression of pattern recognition receptors, proinflammatory cytokine receptors, and inflammatory mediators in these fibroblasts, together with their *in vitro* responses to cytokines, has suggested their direct roles in innate and adaptive host defense and inflammation in RDEB. However, how this inflammatory fibroblast subset might contribute to pathological progression in the RDEB skin, particularly during malignant transformation, remains to be further elucidated.

Fibroblasts have been well known to possess high degrees of plasticity, which allows them to respond to environmental cues under different stress and pathological conditions. Bioinformatic trajectory analysis and anatomic localization examination suggested that inflammatory fibroblasts and myofibroblasts are separate subpopulations, and their induction may have been contributed by different cocktails of cytokines that have been demonstrated to fluctuate over time in RDEB skin.

In this study, we identified IL-1α as the most robustly elevated proinflammatory cytokine in RDEB mice from birth to early adulthood, except for around 1 week of age. IL-1α has been demonstrated to be constitutively present intracellularly in keratinocytes under normal conditions and is released extracellularly upon hypoxic challenge or when undergoing necrosis, activating innate immunity (76). Consistent with this, fibroblasts expressing IL-1R1 were often found to be localized close to the dermal–epidermal junctions where blisters formed. *In vitro* studies further demonstrated that IL-1α simulated IL-1R1 expression on fibroblasts. One of the important differences between IL-1α and IL-1β is that IL-1α is active as a precursor, while IL-1β requires cleavage by caspase-1 following inflammasome formation. We postulate that IL-1α released from damaged epidermal cells triggered the first wave of inflammatory responses during the perinatal period in RDEB. During this innate immune cell response, IL-1α acts on fibroblasts, dermal macrophages, and Langerhans cells, upregulates chemoattractant proteins to recruit neutrophils and other immune cell types, and induces the production of inflammatory cytokines such as IL-6 and TNF. Accordingly, our data demonstrate that RDEB mouse skin in the subsequent 2 weeks undergoes significant elevation of IL-1α, TNF, IL-6, TGF-β1, and IFN-γ and activation of JAK-STAT signaling. It is also within this time frame that TGF-β-induced pSmad2/3 signaling was significantly activated in RDEB mice (29). These cytokines may have overlapping but divergent roles in inducing inflammatory or myofibroblastic phenotypes in RDEB fibroblasts.

Our *in vitro* analysis suggested that the expression of PDPN and ST2, two signature genes in the RDEB inflammatory fibroblast subset identified by scRNAseq, could be stimulated by IL-1α, TNF, and IFN-γ independently, although individual heterogeneity and species discrepancy were observed. It is interesting to note that while PDPN was a robust responder to stimuli in both human and mouse systems (with significant upregulation in RDEB patient-derived or a trend of upregulation in RDEB mouse-derived fibroblasts as compared to their normal controls), the ST2 secretion from human fibroblasts appeared to be more tightly controlled. We postulate that the discrepancy in ST2 expression between human and mouse fibroblasts could be due to interspecies variation, which may have implications for the different roles ST2 plays in each species. As indicated earlier, PDPN is normally expressed on lymphatic endothelial cells and fibroblastic reticular cells to promote blood–lymph separation during development by activating the C-type lectin receptor (CLEC-2) in platelets (77). The expression of PDPN on stromal cells is also essential for the migration of CLEC-2^+^ dendritic cells to lymph nodes to initiate an immune response (77). The pathological involvement of PDPN has often been linked to cancer due to its expression on tumor cells, CAFs, or tumor-associated macrophages, which correlates with a poor prognosis. Recent studies have demonstrated that ectopic expression of PDPN in fibroblasts increased their migratory ability and affected endothelial network formation (78). Interestingly, coexpression of PDPN and sST2 in fibroblasts has not been reported in the literature. sST2, a truncated form of membrane-bound ST2 (ST2L), has been demonstrated to be secreted and function as a decoy receptor for IL-22/ST2L signaling. It is an established prognostic biomarker for cardiovascular diseases, nonrelapse mortality, and severe graft-versus-host disease after hematopoietic stem cell transplantation (79, 80). However, how sST2 is regulated is poorly understood. In this study, RDEB patient-derived fibroblasts exhibited a high degree of heterogeneity in their secretion of sST2, and normal control human fibroblasts had no secretion of sST2 under all the test conditions. It is possible that human fibroblasts may need to be stimulated with a combination of proinflammatory cytokines to secrete sST2. Future studies will be designed to explore this possibility and investigate gene expression and epigenetic modification that may underlie the differential responses of RDEB patient-derived and RDEB mouse fibroblasts to proinflammatory cytokines. Nevertheless, the ability of the mouse fibroblasts to secrete sST2 in response to cytokine stimulation validated the results from the scRNAseq data.

Collectively, our studies demonstrate that the molecular changes endowing an overall immune suppressive and tumor-promoting environment in RDEB occur early in life and are likely imposed by IL-1-initiated cascades that precede TGF-β signaling. Moreover, the MyD88/IL-1R axis, downstream of IL-1 signaling, has been demonstrated to regulate PD-1/PD-L1 expression in melanoma-associated immune cells (81), again in line with the PD-1/PD-L1 expression in RDEB mouse immune cells. Although these changes may be the body’s natural defense in responding against infection and promoting wound healing, repeated inflammation may reprogram the tissue microenvironment into one with compromised immune surveillance, which favors tumor growth. Therefore, while therapeutic approaches targeting TGF-β and JAK-STAT signaling are in active pursuit to suppress fibrosis and cSCC development in RDEB (8, 28, 82–85), strategies targeting more upstream events, for example, IL-1 signaling, and in combination with antagonists to other cytokines or signaling axis such as PD-1/PD-L1, may also be desired to diminish unwanted inflammation and inflammatory tissue priming. Moreover, multiple targets notorious for their association with other disease conditions were identified in our studies, such as TSLP, IL7R, PDPN, sST2, IL-11, SAA3, C3, and CC and CXC chemokines, which may serve as novel targets for RDEB therapeutic development.

This study reveals only the tip of the iceberg of the RDEB dermal microenvironment. We only described the scRNAseq results of fibroblasts, keratinocytes, and immune cells; however, the other cell types are also noteworthy. The full scRNAseq data are accessible in a browsable interface at https://cellxgene.cziscience.com/collections/f5af7a2f-ab4c-4728-829e-48efb9562105, in addition to other resources described in the Data Availability section for those interested in exploring this dataset further.

A major limitation of this study is that the landscape we provided here represents an early phase of RDEB disease progression. We only sampled one timepoint for scRNAseq and performed the kinetic analysis with the mice before their young adulthood due to the shortened RDEB mouse lifespan. We also noticed a high degree of heterogeneity in this study. One KO sample in our scRNAseq exhibited a much higher differential expression than the other sample. Accordingly, large variations in symptomatology and cytokine levels were observed among all the RDEB mice we studied, and heterogeneity was also observed in the RDEB patient-derived fibroblasts.

The pathological progression from blistering and inflammation to dermal fibrosis and malignant transformation has rendered RDEB a unique experimental model for investigating the mechanisms underlying fibroinflammatory disease and tumorigenesis. Our study serves as an example of the amount of complexity that can be uncovered through scRNAseq, which could potentially provide a frameshift in the understanding of RDEB along with other conditions.

## Data availability statement

The datasets presented in this study can be found in online repositories. The names of the repository/repositories and accession number(s) can be found below: GSE222250 (GEO).

## Ethics statement

Ethical approval was not required for the studies on humans in accordance with the local legislation and institutional requirements as RDEB patient-derived fibroblasts were obtained through MTA and IRB approved, and healthy donor cells were commercially available. The animal studies were approved by Kellie A Elson, New York Medical College. The studies were conducted in accordance with the local legislation and institutional requirements. Written informed consent was obtained from the owners for the participation of their animals in this study.

## Author contributions

YL, MSC, AI, and HZ conceived the study. MA-C, HZ, ES, DO, BH, JP, RK, WL, MT, YC, and YL performed animal experiments, single-cell library preparation, cytokine quantitation, flow cytometry analysis, *in vitro* cell culture, and histochemical staining. AA, OI-S, and AI performed computational analysis and interpreted the results. MA-C, AA, OI-S, AI, YL, and MSC wrote the manuscript. YL and AI codirected the study. All the authors reviewed and accepted the manuscript. All authors contributed to the article and approved the submitted version.

## Glossary

**Table:**

APC	antigen presenting cells
CAF	cancer-associated fibroblast
C7	type VII collagen
C7hypo	C7 hypomorphic
cSCC	cutaneous squamous cell carcinoma
CHFB	chondrogenic fibroblasts
ECM	extracellular matrix
iCAF	inflammatory cancer-associated fibroblast
FB	fibroblasts
GO	gene ontology
IFN-γ	interferon gamma
IHC	immunohistochemical
IL	interleukin
IL1R1	IL1 receptor 1
IL-7R	cnterleukin-7 receptor
JAK-STAT	Janus kinases-signal transducer and activator of transcription proteins
KO	knockout
KRT	keratinocytes
LEC	lymphatic endothelial cells
LIF	leukemia inhibitory factor
LYM	lymphocytes
MC	mast cells
MFI	median fluorescent intensity
myCAF	myofibroblastic cancer-associated fibroblast
NF-κB	nuclear factor kappa-light-chain-enhancer of activated B cells
NEU	neutrophils
PAGA	partition-based graph abstraction
PD-1	programmed death protein 1
PD-L1	programmed death-ligand 1
PDPN	podoplanin
PVC	perivascular cells
RDEB	recessive dystrophic epidermolysis bullosa
WT	wild type
SCH	Schwann cells
scRNAseq	single-cell RNA sequencing
SMA	smooth muscle actin
ST2	suppression of tumorigenicity 2
ST2L	a membrane-bound form of ST2
sST2	soluble form of ST2
TCF-1	T-cell factor-1
TGF	transforming growth factor
TNF	tumor necrosis factor
TSLP	thymic stromal lymphopoietin
TSLPR	thymic stromal lymphopoietin receptor
UMAP	uniform manifold approximation and projection
VEC	vascular endothelial cells
