# Supplementary material for: Inflammation-mediated fibroblast activation and immune dysregulation in collagen VII-deficient skin
Source: Front Immunol. 2023 Sep 20;14:1211505. doi: 10.3389/fimmu.2023.1211505 (PMC10557493; doi:10.3389/fimmu.2023.1211505)
Supplement: Supplementary file 1 [file DataSheet_1.zip › Supplementary Methods and Figures.docx]

**Supplementary Materials for**

Inflammation-mediated fibroblast activation and immune dysregulation in collagen VII deficient skin

Morgan Anderson-Crannage & Alex M. Ascensión *et al.*

Corresponding author: Mitchell S. Cairo, [Mitchell_Cairo@nymc.edu](mailto:Mitchell_Cairo@nymc.edu); Ander Izeta, [ander.izeta@biodonostia.org](mailto:ander.izeta@biodonostia.org); Yanling Liao, [Yanling_Liao@nymc.edu](mailto:Yanling_Liao@nymc.edu)

**The file includes:**

Supplementary Method

Supplementary figures S1-S7

List of Data file S1-S6

**Supplementary Methods**:

**Basic processing**

Single-cell analysis was performed using Scanpy (v1.9.2) (*1*). Each of the samples was initially processed independently. QC on cells was performed by calculating the percentage of mitochondrial reads mapping per cell, the number of genes as well as its log(x+1) form. Then, individual thresholds were set as follows: for sample KO1, cells with more than 5.8 and less than 8.0 log(x+1) genes by counts were retained; and for samples KO2, WT1 and WT2, the threshold was 6.5 < log(1+x) < 8.0. Cells with 10% or fewer reads mapping to mitochondrial genes were retained. Then, data were normalized using the *sc.pp.normalize_total()* function, and log1p transformed.

PCA was calculated using the *sc.pp.pca()* function with 30 components. *k*NN graph was calculated using *sc.pp.neighbors()* function, setting the number of neighbors as $\left[ \sqrt{n}/2 \right]$, $n$ being the number of cells of the dataset, and $\left[ \right]$ indicating the integer part of that number; using *cosine* metric. Feature selection was performed using *triku* (v2.1.6) (*2*). UMAP (v.0.4.6) was run using *umap-learn* library implementation in *sc.tl.umap()* with *min_dist* values between 0.3 and 0.5 depending on the dataset (*3*). Clustering was performed with *leiden* using a high resolution value (11-13) for the cluster assignment algorithm, using *sc.tl.leiden()* function (*4*). Then, the cell assignment algorithm described in the following section is run to assign each of the major cell types to the leiden clusters. The processing in this paragraph is also repeated in merged datasets or cell type-specific datasets.

After each sample was processed, all samples were merged using *harmonypy* (v.0.0.5) integrated in Scanpy with the function *sce.pp.harmony_integrate()* with parameters *max_iter_harmony=50*, and *sigma=0.05* (*5*).

The dictionary of cell types and markers used for this step was the following: *Keratinocyte Krt5+* (Krt5, Fgfr2, Lamb3, Col7a1, Gm17851), *Keratinocyte Krt10+* (Krt10, Krt1, Them5, Endou, Klk8), *Keratinocyte Lor+* (Lor, Nccrp1, Trex2, Lce1a1, Lce1b), *Keratinocyte Tbx1+* (Dusp6, Slc7a8, Tbx1, Shisa2, Ucp2), *Keratinocyte Krt28+* (Krt28,Dlx3, Krt25, Krt27, Krt71), *Keratinocyte Krt75+* (Gjb2, Wnt11, Krt75, Fzd5, Fads3), *Keratinocyte Defb6+* (Krt79, Defb6, Atp6v1c2, Nebl, Teddm3), K*eratinocyte Anln+* (Anln, Prc1, Cdk1, Cenpf, Dnph1), *Keratinocyte Cidea+* (Cidea, Ldhb, Aadac, Bex1, Pparg), *Fibroblast Cxcl12+* (Cxcl12, Htra3, C1s1, Lol, Cygb), *Fibroblast Thbs4+* (Thbs4, Spon2, Fmod, Ptgis, Cilp2), *Fibroblast Cxcl1+* (Cxcl1, Tnfaip6, Ccl7, Has1, Cxcl10), *Fibroblast Clec3b+* (Clec3b, Fbn1, Pi16, Scara5, Ugp2), *Fibroblast Col8a1+* (Col8a1, Eid1, Arap1, Gpr153, Igfbp2), *Fibroblast Coch+* (Coch, Crabp1, Fbn2, Emid1, Wfdc1), Fibroblast Rab37+ (Rab37, Col22a1, F13a1, Htra4, Tspan15), *Fibroblast Chf+* (Cfh, Alpl, Lifr, Sp7, Spp1), *Fibroblast Ptgs2+* (Il1rl1, Ptgs2, Nr4a2, Gxylt2, Lum), *Fibroblast Serpine2+* (Serpine2, Shox2, Wif1, Gm48159, Col23a1), *Chondrogenic fibroblast* (Col9a1, Col9a2, Scrg1, Hapln1, Trpv4), *Vascular endothelial cell* (Pecam1, Cldn5, Cdh5, Ptprb, Tie1), *Lymphatic endothelial cell* (Mmrn1, Ccl21a, Prox1, Lyve1, Flt4), *Perivascular cell Inpp4b+* (Rgs5, Myh11, Aoc3, Inpp4b, Mrvi1), *Perivascular cell Il6+* (Rgs5, Myh11, Il6, Procr, Ngf), *Schwann cell* (Prx, Mbp, Mpz, Ncmap, Cldn19), *Glial cell* (Gfra3, Plp1, Scn7a, Cdh19, Adam23), *Melanocyte* (Pmel, Mlana, Dct), *Skeletal muscle* (Msc, Myod1, Cdh15, Peg3, Dag1), *Red blood cell* (Hba-a1, Hbb-bt, Hbb-bs, Car2, Rhd), *T cell* (Cd3d, Cd3e, Ifngr1, Klf2, Cd27), *B cell* (Rrm2, Rpa3, Cd79b, Dntt, Cd79a), *Plasma cell* (Ighm, Igkc, Cd79b, Iglc1, Iglc2), NK cell (Cd3d, Cd3e, Nkg7, Klrk1, Trdv4), *Macrophage* (C1qa, C1qc, Wfdc17, Pf4, Folr2), *Monocyte* (Wfdc17, Csf1r, F10, Ly6c2, Gsr), *Neutrophil* (S100a8, S100a9, Camp, Ltf, Chil3), *Neutrophil** (S100a9, Acod1, Il1f9, Rhov, Stfa2l1), *Dendritic cell* (Cd209a, Irf5, Plbd1, Aif1, Cd209d), *Langerhans cell* (Cd207, Mfge8, Cd74, Il1r2, Tnfaip2), *Mast cell* (Cpa3, Cyp11a1, Cma1, Mcpt4, Tpsb2).

Some of the populations, like keratinocytes or fibroblasts, were determined with a number of different markers because minor populations would sometimes not be assigned. In order to obtain the final merge population list (KRT, FB, CHFB, VEC, LEC, PVC, SCH, MC, LYM, NEU, APC) populations were renamed as follows: all populations beginning with *Keratinocyte* were merged into KRT; all populations beginning with *Fibroblast*, *Melanocyte* and *Skeletal muscle* were merged into FIB; *Chondrogenic fibroblast* was renamed to CHFB; *Vascular endothelial cell* was renamed to VEC; *Lymphatic endothelial cell* was renamed to LEC, *Perivascular cell Inpp4b+* and *Perivascular cell Il6+* were merged into PVC; *Schwann cell* and *Glial cell* were merged to SCH; *Mast cell* was renamed to MC; *T cell*, *B cell*, *NK cell* and *Plasma cell* were merged to LYM; *Neutrophil* and *Neutrophil** were merged to NEU; and *Macrophage*, *Monocyte*, *Dendritic cell*, and *Langerhans cell* were merged to APC.

### Cell assignment algorithm

The aim of this algorithm is to, given a dictionary of populations and their respective markers, generate a mapping between the clusters of the dataset and the populations. For each population, a matrix of shape the number of cells in the AnnData by the number of markers of that category, $M_{0}$, is created.

For each gene in $M_{0}$, the gene expression array is multiplied by the neighbour matrix to produce a matrix that contains the expression of the gene in each cell and in its neighbours. The aim of this product is to reinforce the expression of local genes. This *k*NN matrix is divided by the number of neighbours, and the result is stored in the corresponding column of $M_{0}$.

Once $M_{0}$ has been completed, the mean is extracted, column-wise. This produces a column matrix with the mean *k*NN values across genes, $m_{1}$. We create the matrix $M_{1}$, resulting of concatenating $m_{1}$ matrices with all populations. Therefore, the $M_{1}$ shape is the number of cells in the AnnData by the number of populations.

The cluster labelling from leiden is added to $M_{1}$, and this information is collapsed into a $M_{2}$ matrix, whose dimension is the number of clusters by the number of populations. To collapse the information of several cells from one cluster in $M_{1}$ into one number in $M_{2}$, the percentile defined in the *quantile_gene_sel* argument, the CV, or the maximum value is computed.

Lastly, for the $M_{2}$ matrix with the selected collapsing function, the assigned population is the one with the highest value across clusters. It is possible to select more than one population as the best for each cluster. To do that, *intermediate_states* argument is set to *True* and the populations with a smaller difference to the best population than the one defined in the argument *diff* are selected. If none of the populations achieves a minimum value established in *min_score* the population assigned to the cluster is undefined.

### Analysis of subtype cells

Each of the major populations in this analysis (Fibroblasts, Keratinocytes and Immune cells) was extracted into a separate AnnData for analysis. Fibroblasts were isolated as clusters beginning with *Fibroblast*, Keratinocytes as clusters beginning with *Keratinocyte*, and Immune cells as MC, LYM, NEU, APC types.

The processing of these samples consisted of (1) filtering genes with *sc.pp.filter_genes(adata, min_counts=1)* to remove genes that are expressed in other cell types but not in fibroblasts, (2) *sc.pp.pca* with *n_comps=50*, (3) *scp.pp.harmony_integrate* with *max_iter_harmony=50*, (4) *sc.pp.neighbors* with *n_neighbors*=$\left[ \sqrt{n}/2 \right]$ and *metric=cosine*, and (5) *tk.tl.triku()* for feature selection. Once features were selected, steps 2, 3, and 4 were repeated.

Afterwards, cell subtypes were assigned to each cell type using secondary dictionaries with more genes per category, and some category modifications compared to the dictionary used with the whole dataset. The list of genes was cut up to 7 genes.

The subtypes used for fibroblast characterization were: *FB Pdpn+Il1rl1+*: (Pdpn, Sod2, Il1rl1, Mif, Twist2, Pgk1, Gm29408), *FB Msc+Itga7+*: (Bok, Lrrc15, Msc, Itga7, Etl4, Mcam, Ednrb), *FB Serpine2+Col23a1+*: (Serpine2, Shox2, Wif1, Col23a1, Gm48159, Cd24a, Nav2), *FB Cxcl1+Ccl2+*: (Cxcl1, Ccl7, Has1, Cxcl2, Tnfaip6, Cxcl10, Ccl2), *FB Clec3b+Ly6a+*: (Clec3b, Tnxb, Ly6a, Cd248, Ecm1, Tppp3, Islr), *FB Rab37+Col22a1+*: (Rab37, Col22a1, F13a1, Htra4, Tspan15, Rgcc, Hbegf, Cd55), *FB Cilp2+Acan+*: (Cilp2, Chad, Egfl6, Comp, Fmod, Tnmd, Ecrg4), *FB Lox+Adam33+*: (Mfap4, Lox, Adam33, Eln, Pdgfrl, Ccn5, Fbln5), *FB Coch+Emid1+*: (Coch, Emid1, Gldn, Ntn5, Tnmd, Col16a1, Col11a1), *FB Cfh+Spp1+*: (Cfh, Alpl, Lifr, Sp7, Spp1, Runx2, Cp).

The subtypes used for keratinocyte characterization were: *KRT Krt27+*: (Krt25, Krt27, Krt71, Tchh, Prss53, Ttyh2, Crnn), *KRT Nkd2+*: (Krt73, Msx2, Nkd2, Ctsc, Crym, Fbp1, Dusp2), *KRT Krt35+*: (Krt35, Krt36, Rexo2, Mt4, Gm49425, Msx1, S100a3), *KRT Shisa2+*: (Barx2, Krt6a, Sprr1a, Rnase12, Krt14, Gja1, Krt6a), *KRT Il11ra1+*: (Il11ra1, Col16a1, Tagln, Slc7a8, Cxcl14, Bgn, Vdr), *KRT Id3+*: (Id3, Mt2, Fos, Mt1, Ier2, Krt17, Slc3a2), *KRT Anln*+: (Anln, Prc1, Cdk1, Cenpf, Dnph1, Cdca8, Birc5), *KRT Krt5+*: (Krt5, Igfbp2, Ly6a, Sparc, Cdh13, Il1r2, Efemp1), *KRT Ifi202b+*: (Krt5, S100a10, Eif5, Serpinb2, Ifi202b, Cxcl16, Fosl1), *KRT Krt10+*: (Krt1, Krt10, Mt4, Lgals3, Acsl1, Chit1, Endou), *KRT Krt78+*: (Krt78, Dkkl1, Gm94, Skint5, Klk5, Klk8, Oas1f), *KRT Lor+*: (Lor, Nccrp1, Trex2, Lce1a1, Lce1b, Flg, Lce1f), *KRT Defb6+*: (Krt79, Krt17, Cst6, Ly6g6c, Defb6, Defb1, Klk7), *KRT Sprr1b+*: (Krt16, Sprr1b, Sprr1a, Asprv1, Ehf, Sbsn, Krt80), *KRT Cidea+*: (Mgst1, Cidea, Ldhb, Acsbg1, Pparg, Bex1, Krt79), KRT Cd74+: (Cd74, H2-Eb1, H2-Aa, Vim, Tyrobp, Mfge8, Alox5ap), *KRT Krt75+*: (Krt17, Tm4sf1, Gjb2, Wnt11, Slc39a6, Krt75), *KRT Gpx2+*: (Gpx2, Ly6g6c, Krt6a, Cpm, Cryab, Fads3, Pinlyp), *KRT Fxyd1+*: (Sparc, Vim, Lgals1, Emp3, Crip1, S100a4, Col6a1), *KRT Myh11+*: (Tagln, Col4a2, Col4a1, Acta2, Myh11, Igfbp2, Rbpms), *KRT Krt18+*: (Krt18, Krt9, Cldn3, Cystm1, Wfdc18, Ceacam1, Wfdc2) .

The subtypes used for immune characterization were: *αβT*: (Tnfrsf18, Cxcr6, Ccr2, Ifngr1, Cd52, Rora, Il7r), *γδT*: (Nkg7, Ctsw, Cd7, Rab4a, Ctla2a, Rgs2, Cd3d), *Act. Neu*: (Acod1, Il1f9, Csta3, Rhov, Stfa2l1, Ccl3, Hcar2), *Neu*: (Camp, Ltf, Chil3, S100a8, S100a9, Tinagl1, Stfa1), *Act. LC*: (Fscn1, Cd200, Il4i1, Pkib, Wnk1, Cd74, Cst3), *LC*: (Mfge8, Cd207, Cldn1, Pgf, Cd74, H2-Aa, H2-Eb1, Nr4a3), *Mφ/DCA*: (Nos2, Saa3, Arg1, Wfdc17, Csf1r, F10, Ly6c2), *Mφ/DCB*: (Pf4, C1qb, Igf1, Grn, Dab2, C1qc, Apoe), *Mφ/DCC*: (Cd209a, Cd209d, Slc39a14, Plbd1, Aif1, Cd74, Clec4e), *MC*: (Cma1, Tpsb2, Mcpt4, Fdx1, Hs3st1, Cd55, Tpsab1), *Bas*: (Mcpt8, Cyp11a1, Cd200r3, Ifitm1, Ero1l, Ccl6, Ccl3), *BC*: (Vpreb3, Cd79a, Cd79b, Chchd10, Ighm,Stmn1, Igll1), *PC*: (Iglc3, Ms4a1, Iglc2, Ighm, Igkc, Cd79a, Ly6d).

### Gene ontology (GO) term scoring

Scores for four biological processes (hypoxia, glycolysis, stress and cytokine expression) were computed using the Scanpy function *scanpy.tl.score_genes()*, which averages the expression of a set of genes and subtracts the expression of a randomly sampled set of reference genes. The genes used were selected from specific GO terms. Hypoxia: “cellular response to hypoxia” (GO:0071456). Glycolysis: “glycolytic process” (GO:0006096). Stress: “regulation of translation in response to stress” (GO:0043555) and “regulation of cellular response to oxidative stress” (GO:1900407). Cytokine expression: “positive regulation of inflammatory response” (GO:0050729) and “positive regulation of acute inflammatory response” (GO:0002675).

**Trajectory inference**

PAGA (*6*) was used for trajectory inference of fibroblast subtypes, using ‘fr’ as model. The algorithm was run on all fibroblast datasets, as well as on the set of clusters 0, 1, 2, 3, and 4. Particular visualizations were performed using *diffmap* with the *sc.tl.diffmap()* function (*7*). Pseudotime analysis was performed using Dpt with the *sc.tl.dpt()* function (Haghverdi et al., 2016).

Supplementary figures

Fig. S1


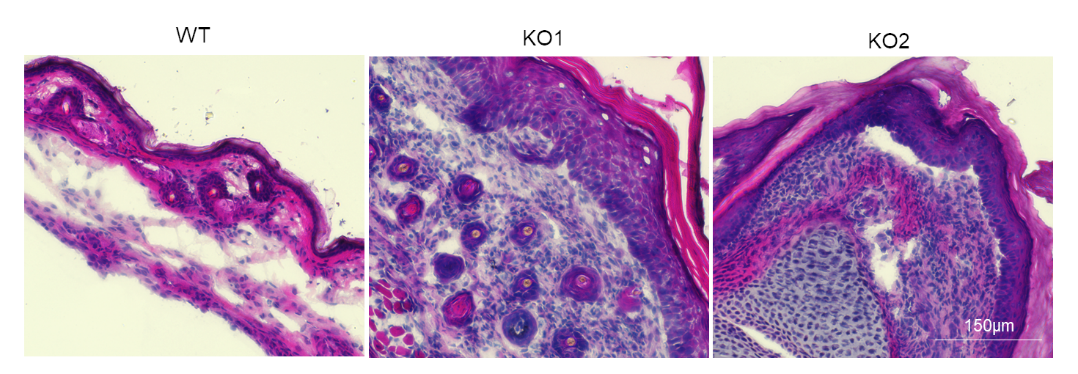


Fig. S1: Hematoxylin and eosin staining of age-matched wild type (WT) paw and hind paws of knockout (KO)1 and KO2 mice utilized for scRNAseq analysis. Scale bar: 150µm.

Fig. S2


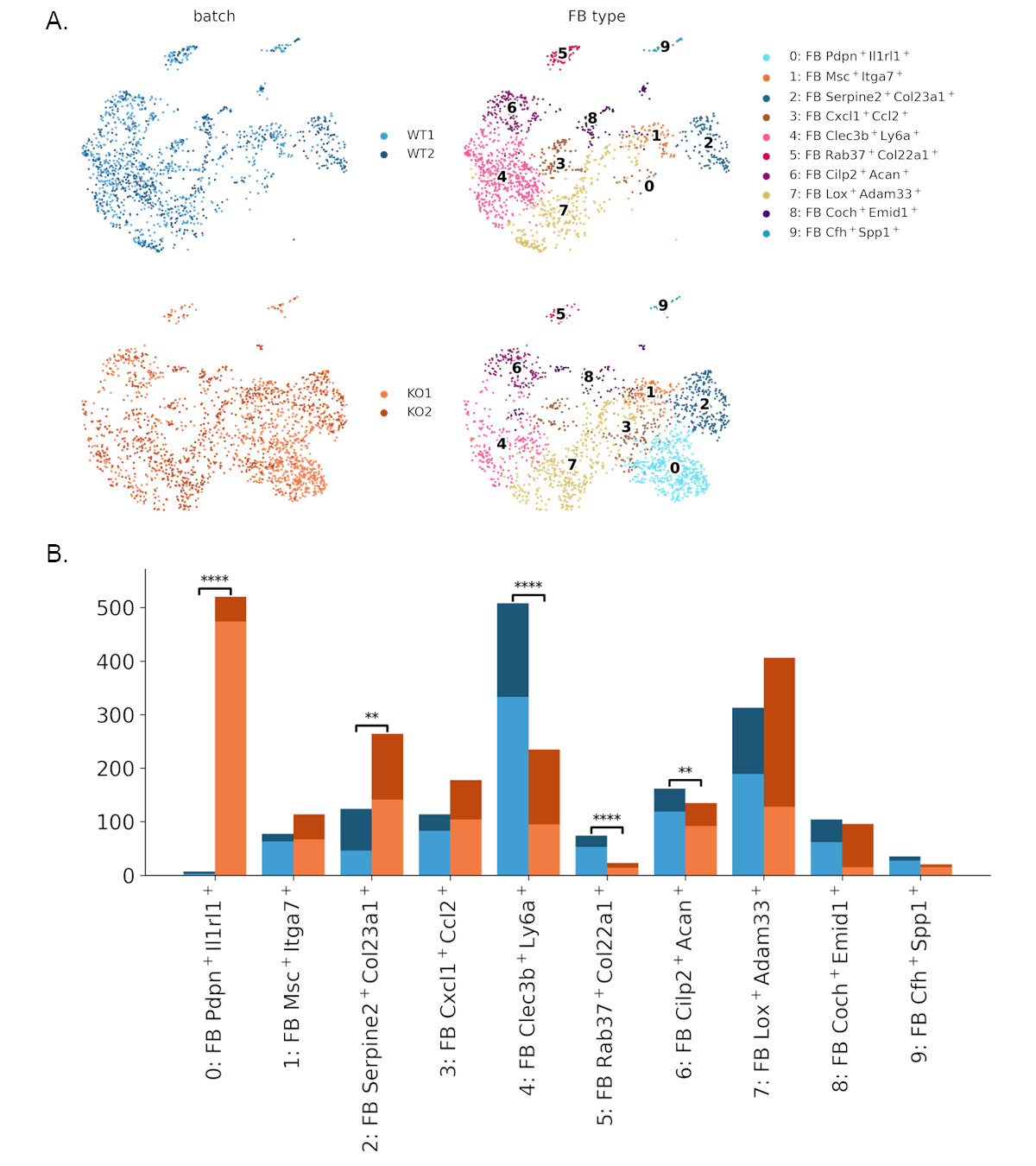


**Figure S2**: Unsupervised clustering of fibroblasts (FB) reveals subclusters unique to KO sample. (A) UMAP plots illustrate transcription differential among WT and KO samples and all fibroblast subclusters identified through unsupervised clustering. (B) Bar graph of total number of cells per cluster and their differences between WT and KO samples. p-value < 0.01 (**) and p-value < 0.0001 (****), χ2 contingency test.

Fig. S3


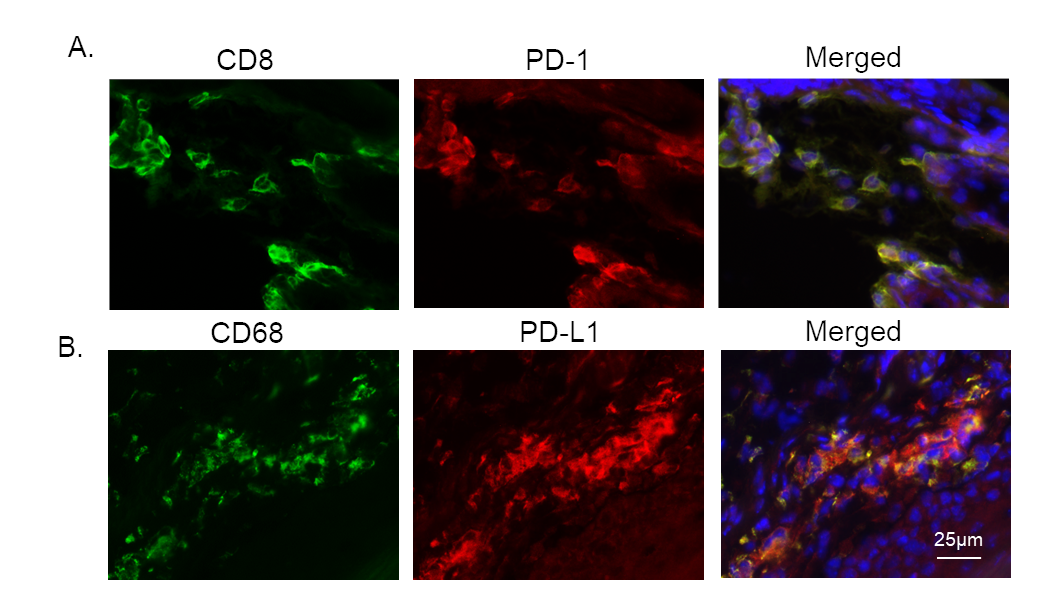


**Figure S3**: PD-1 is expressed on CD8^+^ T cells and PD-L1 is expressed on CD68^+^ macrophage/dendritic cells in the skin of C7^hypo^ mice that had developed mitten deformity. (A-B) Immunohistochemical staining of RDEB mouse skin revealing PD-1 and CD8 colocalization, and PD-L1 and CD68 colocalization. Nuclei stained with DAPI. Scale bar: 25µm.

Fig. S4:


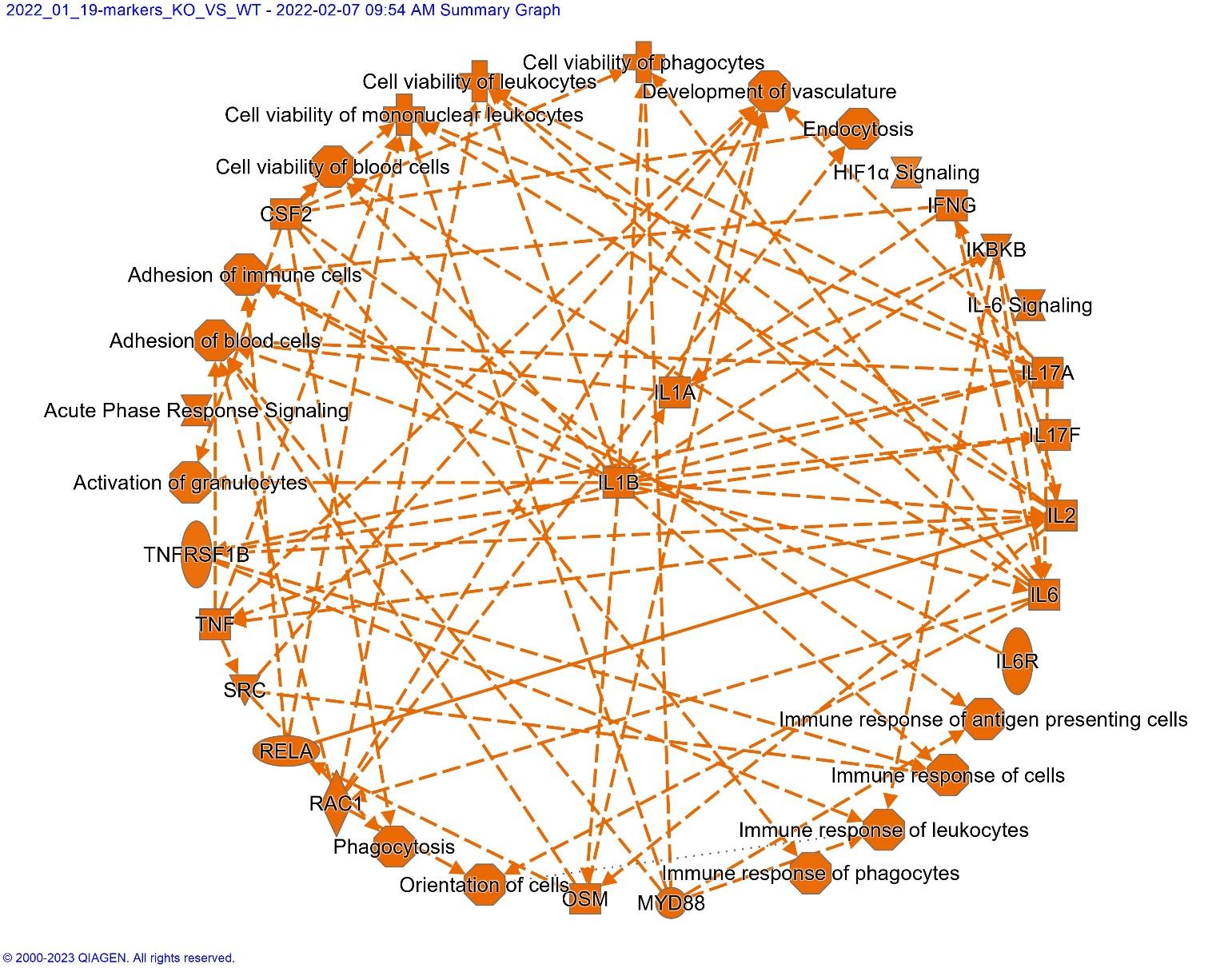


**Figure S4**: Graphic summary of the Qiagen’s Ingenuity Pathway Analysis (IPA) on differential gene expression between KO and WT shows an overall upregulation of immune cell response networks centered on IL-1 in the KO samples.

Fig. S5


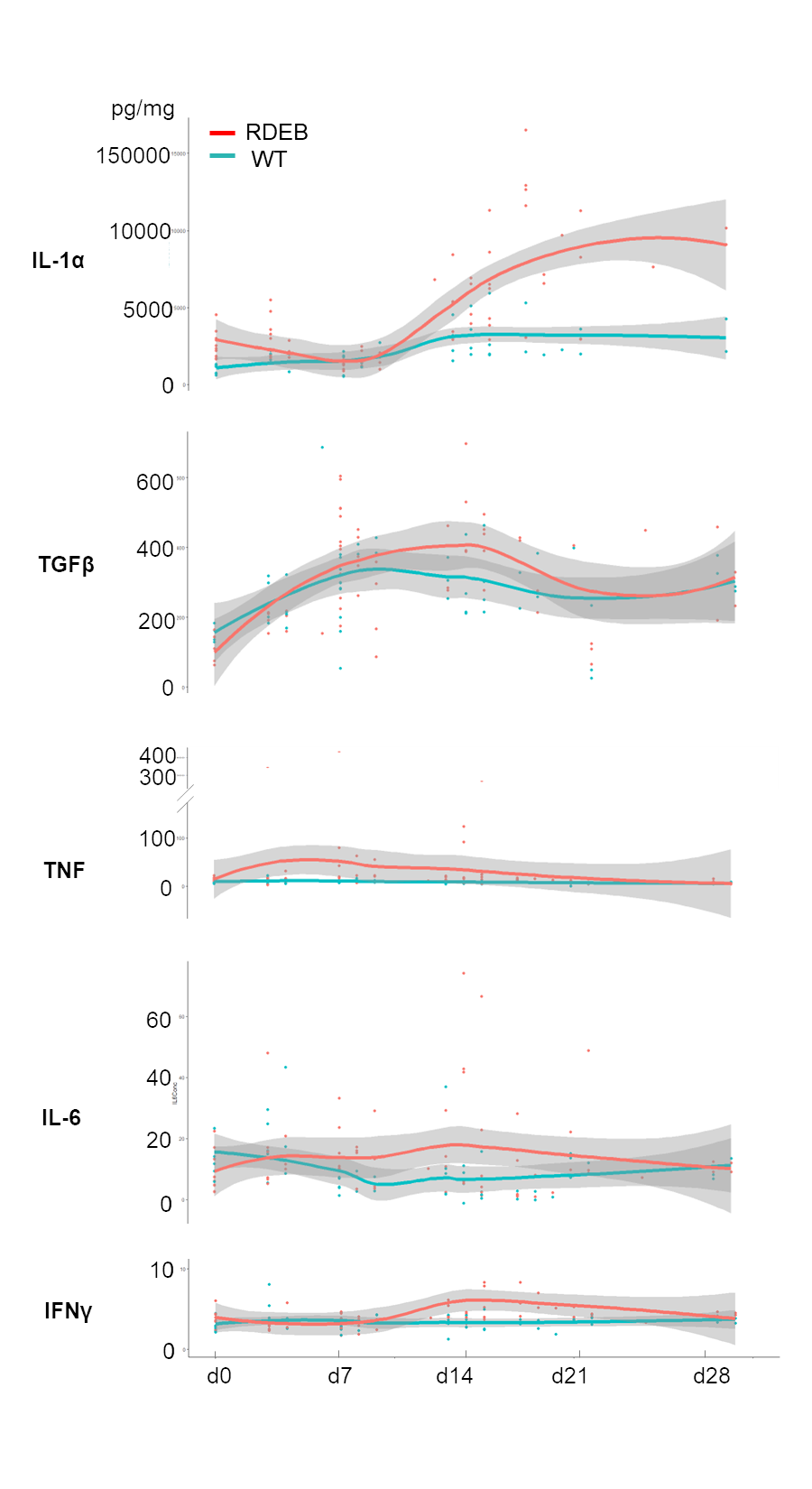


**Figure S5**: IL-1ɑ, TGFβ1, TNF, IL-6, and IFNγ exhibited different dynamics in the WT and RDEB mouse skin within the first month after birth.

Fig. S6


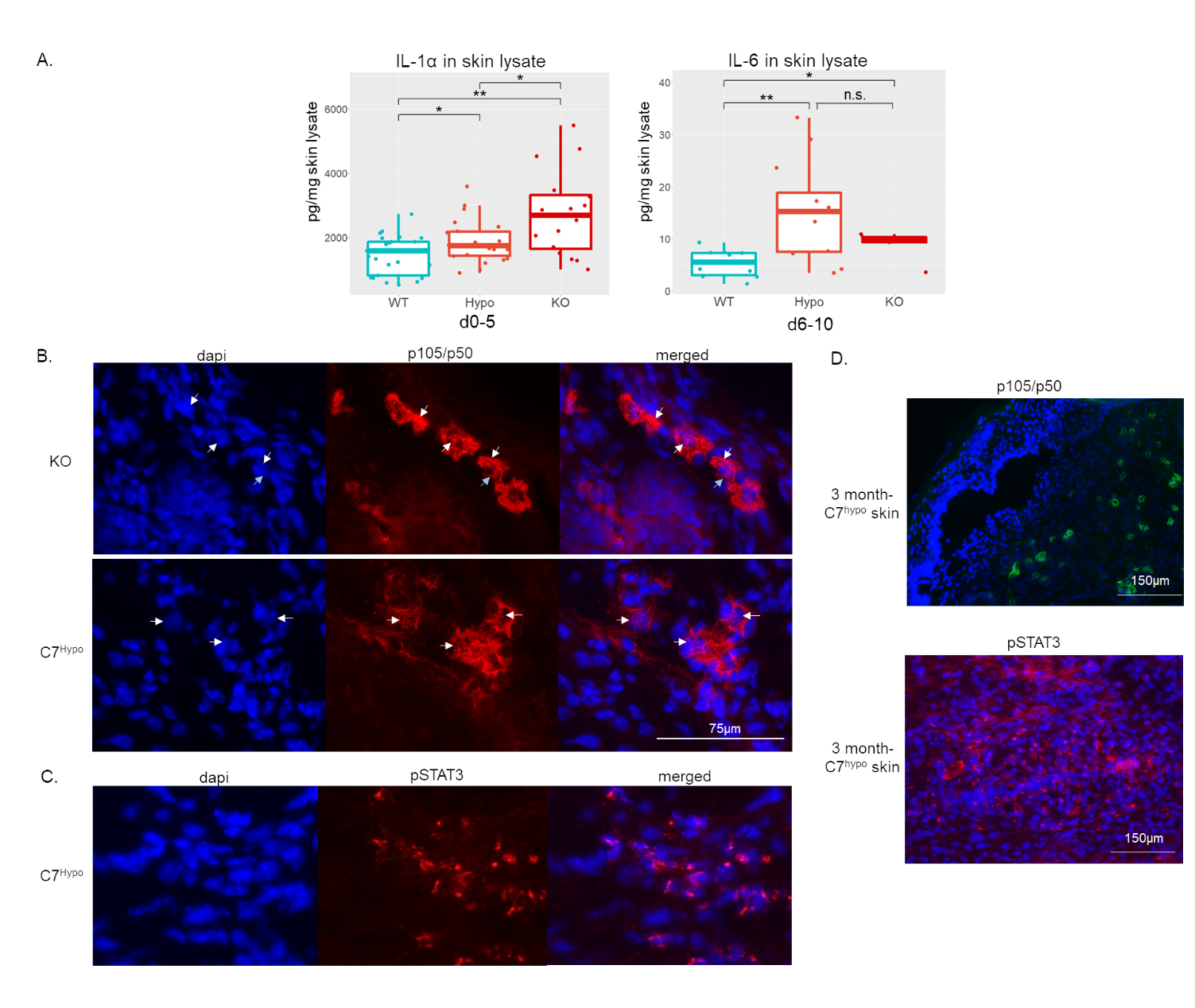


**Figure S6**: Comparison between C7^hypo^ and KO mice. (A) Upregulation in IL-1ɑ and IL-6 expression in C7^hypo^ and KO mice compared to WT mice. IL-1ɑ was also significantly higher in KO mice compared to 8C7^hypo^ mice. IL-6, representative of all the other cytokines, was not statistically different between the KO and C7^hypo^ mice. p-value < 0.05 (*) and p-value < 0.01 (**), unpaired Student t test. (B) KO and C7^hypo^ mouse skin exhibited a similar expression and nuclei localization (shown by arrows) of p105/p50. (C) A magnified image of pSTAT3 staining from Fig. 5F. (D), p50/p105 and pSTAT3 immunohistochemical analysis in 3-month-old C7^hypo^ mouse skin.

Fig. S7


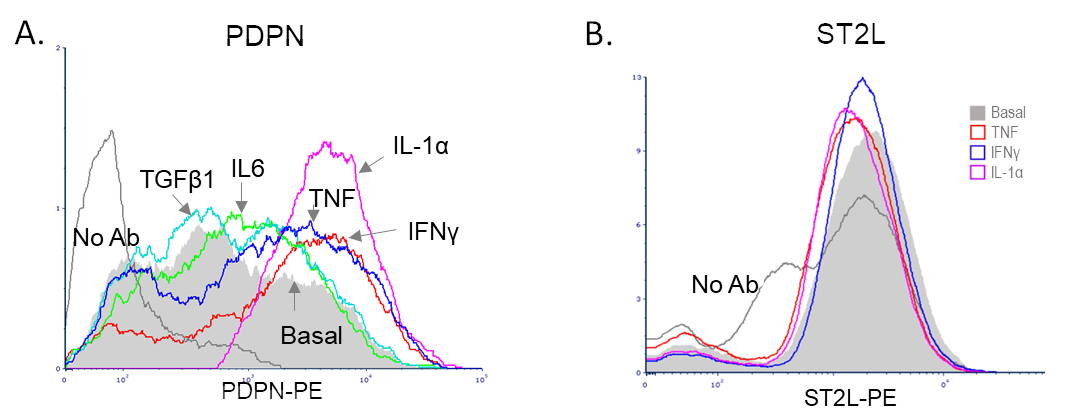


**Figure S7**: Human RDEB patient-derived fibroblasts express elevated PDPN, but not full-length membrane bound ST2 (ST2L) upon inflammatory cytokine stimulation. (A) Representative overlaid plots of the flow cytometry analysis on PDPN expression in human RDEB patient-derived fibroblasts under basal condition, or upon stimulation with IL-1ɑ, TNF, IFNɣ, IL-6, or TGFβ1. (B) Representative overlaid plots of the flow cytometry analysis on ST2L expression in human RDEB patient derived fibroblasts under basal condition, or upon stimulation with IL-1ɑ, TNF or IFNɣ.

**Other Supplementary Materials for this manuscript includes the following**:

**Data file S1**: Top differentially expressed genes among each cell type ordered by p-value. Keratinocytes (KRT), fibroblasts (FB), chondrogenic fibroblasts (CHFB), vascular endothelial cells (VEC), lymphatic endothelial cells (LEC), perivascular cells (PVC), Schwann cells (SCH), mast cells (MC), lymphocytes (LYM), neutrophils (NEU) and antigen presenting cells (APC).

**Data file S2**: Top differentially expressed genes with log2 fold change between KO and WT samples by cell type ordered by p-value (overexpressed in KO). Keratinocytes (KRT), fibroblasts (FB), chondrogenic fibroblasts (CHFB), vascular endothelial cells (VEC), lymphatic endothelial cells (LEC), perivascular cells (PVC), Schwann cells (SCH), mast cells (MC), lymphocytes (LYM), neutrophils (NEU) and antigen presenting cells (APC).

**Data file S3**: Top differentially expressed genes with log2 fold change between WT and KO samples by cell type ordered by p-value (overexpressed in WT). Keratinocytes (KRT), fibroblasts (FB), chondrogenic fibroblasts (CHFB), vascular endothelial cells (VEC), lymphatic endothelial cells (LEC), perivascular cells (PVC), Schwann cells (SCH), mast cells (MC), lymphocytes (LYM), neutrophils (NEU) and antigen presenting cells (APC).

**Data file S4**: Top differentially expressed genes among fibroblast subtypes ordered by p-value.

**Data file S5**: Top differentially expressed genes among immune cells ordered by p-value.

**Data file S6**: Top differentially expressed genes among keratinocyte subtypes ordered by p-value.

1. F. A. Wolf, P. Angerer, F. J. Theis, SCANPY: large-scale single-cell gene expression data analysis. *Genome Biol* **19**, 15 (2018).

2. M. A. A, O. Ibanez-Sole, I. Inza, A. Izeta, M. J. Arauzo-Bravo, Triku: a feature selection method based on nearest neighbors for single-cell data. *Gigascience* **11**, (2022).

3. L. McInnes, J. H. , N. S. , a. L. , Großberger, UMAP: Uniform Manifold Approximation and Projection. *Journal of Open Source Software* **3**, (2018).

4. V. A. Traag, L. Waltman, N. J. van Eck, From Louvain to Leiden: guaranteeing well-connected communities. *Scientific reports* **9**, 5233 (2019).

5. I. Korsunsky *et al.*, Fast, sensitive and accurate integration of single-cell data with Harmony. *Nat Methods* **16**, 1289-1296 (2019).

6. F. A. Wolf *et al.*, PAGA: graph abstraction reconciles clustering with trajectory inference through a topology preserving map of single cells. *Genome Biol* **20**, 59 (2019).

7. R. R. Coifman *et al.*, Geometric diffusions as a tool for harmonic analysis and structure definition of data: diffusion maps. *Proceedings of the National Academy of Sciences of the United States of America* **102**, 7426-7431 (2005).
